# Multivariate Bayesian spatio-temporal P-spline models to analyze crimes against women

**DOI:** 10.1093/biostatistics/kxab042

**Published:** 2021-12-27

**Authors:** Gonzalo Vicente, Tomás Goicoa, María Dolores Ugarte

**Affiliations:** Department of Statistics, Computer Science, and Mathematics, Public University of Navarre, Campus de Arrosadia, 31006 Pamplona, Spain and Institute Institute for Advanced Materials and Mathematics (INAMAT2), Campus de Arrosadia, 31006 Pamplona, Spain; Department of Statistics, Computer Science, and Mathematics, Public University of Navarre, Campus de Arrosadia, 31006 Pamplona, Spain and Institute Institute for Advanced Materials and Mathematics (INAMAT2), Campus de Arrosadia, 31006 Pamplona, Spain and IdiSNA, Health Research Institute of Navarre Recinto de Complejo Hospitalario de Navarra C/ Irunlarrea, 3 31008 Pamplona, Spain; Department of Statistics, Computer Science, and Mathematics, Public University of Navarre, Campus de Arrosadia, 31006 Pamplona, Spain and Institute Institute for Advanced Materials and Mathematics (INAMAT2), Campus de Arrosadia, 31006 Pamplona, Spain and IdiSNA, Health Research Institute of Navarre Recinto de Complejo Hospitalario de Navarra C/ Irunlarrea, 3 31008 Pamplona, Spain

**Keywords:** Bayesian inference, Gender-based violence: INLA, Smoothing, Spatio-temporal patterns

## Abstract

Univariate spatio-temporal models for areal count data have received great attention in recent years for estimating risks. However, models for studying multivariate responses are less commonly used mainly due to the computational burden. In this article, multivariate spatio-temporal P-spline models are proposed to study different forms of violence against women. Modeling distinct crimes jointly improves the precision of estimates over univariate models and allows to compute correlations among them. The correlation between the spatial and the temporal patterns may suggest connections among the different crimes that will certainly benefit a thorough comprehension of this problem that affects millions of women around the world. The models are fitted using integrated nested Laplace approximations and are used to analyze four distinct crimes against women at district level in the Indian state of Maharashtra during the period 2001–2013.

## 1. Introduction

The statistical toolkit for the analysis of spatial and spatio-temporal areal count data has been enriched during recent years with relevant advances in model proposals, algorithms for inference, and the realm of applications (see e.g., Lawson *and others,* 2016; Martínez-Beneito and Botella-Rocamora, 2019). One of the goals of these techniques is smoothing standardized incidence/mortality ratios or crude rates to uncover geographical patterns and temporal trends of the phenomenon under study. These techniques have experienced a great success to study incidence and mortality of different diseases (mainly cancer), and very recently, they have proven their utility to study crimes against women (see Vicente *and others,* 2020a). However, although univariate modeling of several forms of violence against women is very informative, modeling different crimes jointly improves precision estimates and allows computing correlations among them. This is relevant in the research of crimes against women, an extraordinary complex problem where the factors that may be related to the phenomenon are unclear. The problem is even more arduous in India, a country with a wide diversity of traditions, religious beliefs, and social practices, where unraveling potential factors exerting some influence on violence against women is a challenge. Indeed, the multivariate modeling of several crimes is useful to provide correlations between the spatial and temporal patterns of the crimes that may reflect the association with common underlying risk factors and the connections between the different forms of violence on women. This can contribute to narrow down the range of social, religious, or economic characteristics that may be related with the crimes. In this article, we develop P-spline models to analyze four distinct crimes against women at district level in the Indian state of Maharashtra during the period 2001–2013. Namely, rape, assault, cruelty by husband or relatives, and kidnapping and abduction.

The research on multivariate models for spatial count data is now rich, though their use is still limited due to the computational burden and the lack of software that can be adopted by practitioners without advanced computer skills. Most of the research extends univariate CAR models to the multivariate setting. An appealing approach to multivariate modeling is the coregionalization framework (Jin *and others,* 2007; Martinez-Beneito, 2013; MacNab, 2018). However, it may seriously increase computational burden and the so-called M-models (Botella-Rocamora *and others,* 2015) have emerged as an interesting alternative. Recently, Vicente *and others* (2020b) have applied M-based models to the analysis of two forms of crime against women in Uttar Pradesh, India.

When the observed risk variability within responses is high, the use of splines for smoothing risks is an attractive modeling approach (Goicoa *and others,* 2012). Research on multivariate spline models for spatio-temporal count data is not so abundant, and it focuses on multivariate structures to deal with spatial and temporal dependence for one response measured in several time periods. Within the class of P-spline models, MacNab (2007) proposes one-dimensional P-splines to smooth temporal trends, whereas Ugarte *and others* (2010) introduce three-dimensional P-spline models to study the spatio-temporal evolution of mortality risks. In all studies, the structure matrices of space and time are known and only the precision/variance parameters controlling the degree of smoothing have to be estimated (see in addition MacNab and Gustafson, 2007; Ugarte *and others,* 2017). As far as we know, there is no research about multivariate P-spline models for spatio-temporal areal count data in which different responses are analyzed at the same time, and between-response correlation matrices have to be estimated.

The purpose of this article is 3-fold: to propose new models based on P-splines to analyze multivariate spatio-temporal count data; to use the new proposal to study data on crimes against women at district level in the Indian state of Maharashtra over the period 2001–2013, and to implement the methodology in R-INLA to reduce computational burden facilitating the fit of these models to a wide audience.

The rest of the article has the following structure. Section 2 proposes multivariate spatial and temporal P-splines accounting for correlations between the different responses of interest (crimes against women here). Section 3 briefly explains model fitting with R-INLA, hyperprior distributions, and some identifiability issues. Section 4 presents the case study. We close the article with a discussion.

## 2. Multivariate P-spline models

Let us denote by $O_{itj}$ the observed number of cases in area $i=1,\ldots,I$, time $t=1,\ldots,T$, and crime $j=1,\ldots J$. Conditional on the relative risks $R_{itj}$, we assume a Poisson distribution for the number of observed cases

$$
\begin{equation*}
O_{itj}| R_{itj} \sim {\rm Poisson}(\mu_{itj}=E_{itj} \cdot R_{itj}),
\end{equation*}$$

where $E_{itj}$ denotes the expected number of cases computed using indirect standardization as $E_{itj}=n_{it}m_j$. Here, $n_{it}$ is the female population at risk in area $i$ and time $t$, and $m_j$ is the overall rate of crime $j$ in the total area of study (state of Maharashtra in this article) and the whole period. In this article, we propose P-spline models to smooth the spatial and temporal patterns. That is

(2.1) \begin{equation*}\label{risk.individual} \log (R_{itj})= \alpha_j + f_{j}(x_{1i},x_{2i}) + f_{j}(x_{3t}) + \delta_{itj}, \end{equation*}

where $\alpha_j$ is the intercept for the $j$th crime, $x_{1i}$ and $x_{2i}$ are the longitude and latitude of the centroid of the $i$th area; $x_{3t}$ indicates the time point $t$, and $f_{j}(x_{1i},x_{2i})$ and $f_{j}(x_{3t})$ are a smooth spatial surface and a smooth temporal trend respectively for the $j$th crime that are well approximated using P-splines. Finally, $\delta_{itj}$ is the spatio-temporal interaction term for crime $j$. The smooth surface for each crime is specified as ${\mathbf{f}}_j({\mathbf{x}}_1,{\mathbf{x}}_2) = {\mathbf{B}}_s {\boldsymbol{\psi}}^{(j)}$, where ${\mathbf{f}}_j({{\mathbf{x}}}_1,{{\mathbf{x}}}_2)=(f_{j}(x_{11},x_{21}),\ldots,f_{j}(x_{1I},x_{2I}))'$, and $\mathbf{B}_s=\mathbf{B}_2\square \mathbf{B}_1$ is a two-dimensional B-spline basis of dimension $I\times k_1k_2$ arising from the row-wise Kronecker product, denoted by $\square$, of the marginal B-spline bases for longitude, $\mathbf{B}_1$, and latitude $\mathbf{B}_2$ (see Eilers *and others,* 2006). More precisely, the row-wise Kronecker product is defined as $\mathbf{B}_2\Box\mathbf{B}_1=(\mathbf{B}_2\otimes {\bf 1}'_{k_1})\odot({\bf 1}'_{k_2}\otimes \mathbf{B}_1)$, where ${\bf 1}_{k_1}$ and ${\bf 1}_{k_2}$ are column vectors of ones of lengths $k_1$ and $k_2$, respectively; $k_1$ and $k_2$ are the number of columns of ${\bf B}_1$ and ${\bf B}_2$, respectively, and the symbol $\odot$ is the element-wise matrix product. Note that $k_1$ and $k_2$ are the number of columns of the marginal bases $\mathbf{B}_1$ and $\mathbf{B}_2$, respectively, and depend on the number of knots and the degree of the polynomials used to construct these bases. Finally, the vectors ${\boldsymbol{\psi}}^{(j)}=(\psi_1^j,\ldots,\psi_{k_1k_2}^j)'$, $j=1,\ldots,J$ denote the set of coefficients of the spatial B-splines for each crime. To achieve smoothness, the following prior distribution with Gaussian Kernel is marginally considered for the coefficients of the two-dimensional B-splines

(2.2) \begin{equation*}\label{prior.spatial} p({\boldsymbol{\psi}}^{(j)}|\lambda_1,\lambda_2) \propto \exp\left(-\dfrac{1}{2}{\boldsymbol{\psi}}^{(j)'} \mathbf{P}_s {\boldsymbol{\psi}}^{(j)}\right).\end{equation*}

Here, $\mathbf{P}_s= \lambda_1(\mathbf{I}_{k_{2}}\otimes \mathbf{P}_1)+\lambda_2( \mathbf{P}_2 \otimes \mathbf{I}_{k_{1}})$, $\mathbf{P}_1=\mathbf{D}_1' \mathbf{D}_1$, $\mathbf{P}_2 = \mathbf{D}_2' \mathbf{D}_2$, $\mathbf{I}_{k_1}$ and $\mathbf{I}_{k_2}$ are $k_1\times k_1$ and $k_2\times k_2$ identity matrices, $\lambda_1$ and $\lambda_2$ control the amount of smoothing in longitude and latitude, and $\mathbf{D}_1$ and $\mathbf{D}_2$ are difference matrices (of order 1 or 2) of dimension $k_1 \times k_1$ and $k_2\times k_2$, respectively. An in-depth explanation of this penalty can be found in Goicoa *and others* (2016). Similarly, the smooth temporal trend for each crime is specified as $\mathbf{f}_j ( \mathbf{x}_3) = \mathbf{B}_3 {\boldsymbol{\gamma}}^{(j)}$, where now $\mathbf{f}_j( \mathbf{x}_3) = (f_j(x_{31}),\ldots,f_j(x_{3T}))'$, $\mathbf{B}_3$ is the temporal B-spline basis of dimension $T\times k_3$, and $k_3$ depends on the number of knots and the degree of the polynomials in the basis. The vectors ${{\boldsymbol{\gamma}}}^{(j)}=(\gamma_1^j,\ldots,\gamma_{k_3}^j)'$, $j=1,\ldots,J$ represent the coefficients of the temporal B-spline basis for each crime. Here, the following prior distribution with Gaussian kernel is marginally considered

(2.3) \begin{equation*}\label{prior.temporal} p({\boldsymbol{\gamma}}^{(j)}|\lambda_3) \propto \exp\left(-\dfrac{1}{2}{\boldsymbol{\gamma}}^{(j)'} \mathbf{P}_3 {\boldsymbol{\gamma}}^{(j)}\right), \end{equation*}

where ${\mathbf{P}}_3=\lambda_3 \mathbf{D}_3' \mathbf{D}_3$, $\lambda_3$ controls the temporal smoothing and $\mathbf{D}_3$ is a difference matrix (of order 1 or 2) of dimension $k_3\times k_3$. According to Lang and Brezger (2004), we would like to emphasize that the prior distributions in (2.2) and (2.3) are the Bayesian analogues to the difference penalty on the coefficients proposed by Eilers and Marx (1996) to achieve smoothness. This is clear as the matrices ${\mathbf{P}}_l = \mathbf{D}_l' \mathbf{D}_l$, $l=1,2,3$ are the structure matrices of a first (RW1) or second (RW2) order random walk (see Rue and Held, 2005, pp. 95 and 110). Note that (2.2) is the combination of random walk priors on the B-spline coefficients of longitude and latitude (see Belitz and Lang, 2008). For simplicity, we will refer to RW1 or RW2 prior distributions on the coefficients of the spatial and temporal B-splines.

To take account of the potential relationships between the different crimes, correlations among the P-spline coefficients (spatial or temporal) of the different crimes are introduced in the model. Then, the following $J\times J$ covariance matrices between the sets of spatial and temporal P-splines coefficients are assumed

$$
\begin{equation*}
{\boldsymbol{\Sigma}}_{\psi}=(\mathrm{Cov}({\boldsymbol{\psi}}^{(j)},{\boldsymbol{\psi}}^{(k)}))_{j,k=1,\ldots,J},\quad {\boldsymbol{\Sigma}}_{\gamma}=(\mathrm{Cov}({{\boldsymbol{\gamma}}}^{(j)},{{\boldsymbol{\gamma}}}^{(k)}))_{j,k=1,\ldots,J}.\end{equation*}$$

The diagonal elements of ${\boldsymbol{\Sigma}}_{\psi}$ are $({\boldsymbol{\Sigma}}_{\psi})_{jj}=\sigma^2_{\psi j}$, $j=1,\ldots, J$, and the off-diagonal elements are given by $({\boldsymbol{\Sigma}}_{\psi})_{jk}=\rho_{\psi jk}\sigma_{\psi j}\sigma_{\psi k}$, where $\rho_{\psi jk}$ is the correlation between the coefficients of the spatial P-splines for crimes $j$ and $k$. Similarly, the diagonal elements of ${\boldsymbol{\Sigma}}_{\gamma}$ are $({\boldsymbol{\Sigma}}_{\gamma})_{jj}=\sigma^2_{\gamma j}$, $j=1,\ldots, J$, and the off-diagonal elements are expressed as $({\boldsymbol{\Sigma}}_{\gamma})_{jk}=\rho_{\gamma jk}\sigma_{\gamma j}\sigma_{\gamma k}$, where now, $\rho_{\gamma jk}$ represents the correlation between the coefficients of the temporal P-splines for crimes $j$ and $k$.

Finally, ${\boldsymbol{\delta}}^{(j)}=(\delta_{11}^{(j)},\ldots, \delta_{I1}^{(j)},\ldots,\delta_{1T}^{(j)},\ldots, \delta_{IT}^{(j)})'$ is the spatio-temporal interaction for the $j$th crime, $j=1\ldots,J$. For the interaction term, the following prior distribution is assumed

$$
\begin{equation*}
p({\boldsymbol{\delta}}^{(j)}|\tau_j) \propto \exp\left(-\dfrac{1}{2}{\boldsymbol{\delta}}^{(j)'} \mathbf{M}_j \delta^{(j)}\right),
\end{equation*}$$

where $\mathbf{M}_j$ is a precision matrix that can take different forms according to the four types of interactions defined by Knorr-Held (2000). For the Type I interaction, $\mathbf{M}_j=\tau_j \mathbf{I}_{IT}$, where $\mathbf{I}_{IT}$ is the $IT\times IT$ identity matrix, and all the elements of the interaction are independent. In Type II interaction, $\mathbf{M}_j=\tau_j (\mathbf{Q}_{T} \otimes \mathbf{I}_I)$, where $\mathbf{I}_{I}$ is the $I\times I$ identity matrix, and $\mathbf{Q}_{T}$ is the precision matrix of a random walk of first or second order (see Rue and Held, 2005, pp. 95 and 110). This means that the elements of ${\boldsymbol{\delta}}^{(j)}$ are structured in time but not in space. In Type III interactions, the elements of ${\boldsymbol{\delta}}^{(j)}$ are structured in space but not in time. Then, $\mathbf{M}_j=\tau_j (\mathbf{I}_{T} \otimes \mathbf{Q}_I)$, where now $\mathbf{I}_{T}$ is the $T\times T$ identity matrix, and $\mathbf{Q}_I$ is the spatial neighborhood matrix defined as $\mathbf{Q}_{I(im)}=-1$ if areas $i$ and $m$ are neighbors and 0 otherwise, and the ith diagonal element $\mathbf{Q}_{I (ii)}$ is the number of neighbors of the ith area. Finally, in Type IV interaction, the elements of ${\boldsymbol{\delta}}^{(j)}$ are structured in space and time, and $\mathbf{M}_j=\tau_j (\mathbf{Q}_{T} \otimes \mathbf{Q}_I)$. Note that unlike the main spatial and temporal terms, the spatio-temporal interaction is not modelled with P-splines but with conditional autoregressive (CAR) priors. The interaction effect can be seen as a residual term which generally captures a small portion of variability, and hence using three-dimensional P-splines for this term could unnecessarily complicate the model. Similarly, and to simplify the proposal, we do not consider any covariance estructure between the interaction terms of the different crimes, that is $(\mathrm{Cov}({\boldsymbol{\delta}}^{(j)},{\boldsymbol{\delta}}^{(k)}))_{j,k=1,\ldots,J}=\mathbf{I}_J$, where $\mathbf{I}_J$ is a $J\times J$ identity matrix. Our approach is somehow based on the multivariate proposal of Aitchison and Ho (1989) to capture overdispersion in multivariate count data, though these authors do not model spatio-temporal data.

For notation purposes and to incorporate the dependencies between different crimes in the model, we stack the set of spatial and temporal P-splines’ coefficients for each crime one under the other as ${\boldsymbol{\Psi}} = ({\boldsymbol{\psi}}^{(1)'},\ldots,{\boldsymbol{\psi}}^{(J)'})'$ and ${\boldsymbol{\Gamma}}=({\boldsymbol{\gamma}}^{(1)'},\ldots,{\boldsymbol{\gamma}}^{(J)'})'$. Similarly, the interaction terms are rearranged one under the other as ${\boldsymbol{\Delta}}=({\boldsymbol{\delta}}^{(1)'},\ldots,{\boldsymbol{\delta}}^{(J)'})'$. Using this notation, our multivariate P-spline model (2.1) can be expressed in matrix form as

(2.4) \begin{equation*} \label{modelPsplines} \log(\mathbf{R}) = (\mathbf{I}_J \otimes \mathbf{1}_T \otimes \mathbf{1}_I){\boldsymbol{\alpha}} + (\mathbf{I}_J\otimes \mathbf{1}_T \otimes \mathbf{B}_s ) {\boldsymbol{\Psi}} + (\mathbf{I}_J \otimes \mathbf{B}_3 \otimes \mathbf{1}_I){\boldsymbol{\Gamma}} + (\mathbf{I}_J \otimes \mathbf{I}_T \otimes \mathbf{I}_I){\boldsymbol{\Delta}}, \end{equation*}

where ${\boldsymbol{\alpha}}=(\alpha_1,\ldots\alpha_J)'$, ${\mathbf{R}}=(\mathbf{R}'_1,\ldots,\mathbf{R}'_J)'$, $\mathbf{R}'_j= (R_{11,j},\ldots,R_{I1,j},\ldots,R_{1T,j},\ldots,R_{IT,j})$, $j=1,\ldots,J$, and $\mathbf{1}_I$ and $\mathbf{1}_T$ are column vectors of ones of length $I$ and $T,$ respectively. Then, once the between-crime dependencies are incorporated into the model, the resulting prior distributions with Gaussian kernel for the coefficients of the spatial P-splines ${{\boldsymbol{\Psi}}}$, the coefficients of the temporal P-splines ${{\boldsymbol{\Gamma}}}$, and the interaction terms ${\boldsymbol{\Delta}}$ are

(2.5) \begin{eqnarray*} \label{final_priors} p({{\boldsymbol{\Psi}}}|{\boldsymbol{\Sigma}}_{\psi},\lambda_1,\lambda_2) &\propto& \exp\left(-\dfrac{1}{2}{\boldsymbol{\Psi}}' ({\boldsymbol{\Sigma}}_{\psi}^{-1}\otimes \mathbf{P}_s) {\boldsymbol{\Psi}}\right)\nonumber \\ p({{\boldsymbol{\Gamma}}}|{\boldsymbol{\Sigma}}_{\gamma},\lambda_3) &\propto& \exp\left(-\dfrac{1}{2}{\boldsymbol{\Gamma}}' ({\boldsymbol{\Sigma}}_{\gamma}^{-1}\otimes \mathbf{P}_3) {\boldsymbol{\Gamma}}\right)\nonumber \\ p({\boldsymbol{\Delta}}|\tau_1,\ldots,\tau_J) &\propto& \exp\left(-\dfrac{1}{2}{\boldsymbol{\Delta}}' (\mathrm{Blockdiag}(\mathrm{M}_1,\ldots,\mathrm{M}_J)) {\boldsymbol{\Delta}}\right).\end{eqnarray*}

Priors (2.5) have two goals: on one hand the matrices $\mathbf{P}_s$ and $\mathbf{P}_3$ are introduced to produce smooth estimates of the spatial patterns and the temporal trends, respectively, and on the other hand the matrices ${\boldsymbol{\Sigma}}_{\psi}$ and ${\boldsymbol{\Sigma}}_{\gamma}$ cope with the between-crime dependencies. Similarly, the within-crimes remaining spatio-temporal variability is captured through matrices $\mathrm{M}_j$, and no between-crime covariance structure is considered for this term. The priors (2.5) depend on a set of hyperparameters given by the elements of ${\boldsymbol{\Sigma}}_{\psi}$ and $\lambda_1$ and $\lambda_2$ (for ${{\boldsymbol{\Psi}}}$), the elements of ${\boldsymbol{\Sigma}}_{\gamma}$ and $\lambda_3$ (for ${{\boldsymbol{\Gamma}}}$), and $\tau_1,\ldots,\tau_J$ (for ${\boldsymbol{\Delta}}$). It is worth remarking that the $\lambda_3$ parameter in the precision matrix of the temporal P-splines is not identifiable as it can be subsumed in the matrix ${\boldsymbol{\Sigma}}_{\gamma}^{-1}$ (or $\sigma^2_3=1/\lambda_3$ is subsumed in ${\boldsymbol{\Sigma}}_{\gamma}$). Similarly, only the ratio $\lambda_1/\lambda_2$ (or $\lambda_2/\lambda_1$) is identifiable as one of such parameters can be subsumed in the matrix matrix ${\boldsymbol{\Sigma}}_{\psi}^{-1}$. This is clear if one express the precision matrix of the spatial P-splines as $\mathbf{P}_s=\lambda_2\left[\dfrac{\lambda_1}{\lambda_2} (\mathbf{I}_{k_{2}}\otimes \mathbf{P}_1)+(\mathbf{P}_2 \otimes \mathbf{I}_{k_{1}})\right]$. Consequently, we set $\lambda_2$ and $\lambda_3$ equal to 1.

## 3. Model fitting, hyperprior distributions, and identifiability issues

In this article, the models are fitted using INLA (Rue *and others,* 2009) with the $\textsf{R-INLA}$ package (Lindgren and Rue, 2015). INLA has become very popular as it avoids long computing time in comparison to MCMC techniques. INLA relies on integrated nested Laplace approximations and numerical integration for approximate Bayesian inference. It is particularly designed for latent Gaussian models where the latent field is Gaussian, the response is non-Gaussian, and the posterior marginals cannot be obtained in closed form. One of the main advantages of INLA is that many of the models in the literature are already included in the package R-INLA, and others can be implemented by means of generic functions with some extra-programming work. Our multivariate proposal based on P-splines is not directly available in R-INLA and here we implement it using the “$\textsf{rgeneric}$” construction in R-INLA. In particular, we build our model based on the function “$\textsf{inla.rgeneric.IMCAR.model}$” from the R package $\textsf{INLAMSM}$ (https://CRAN.R-project.org/package=INLAMSM) developed by Palmí-Perales *and others* (2021) and designed for fitting multivariate extensions of intrinsic conditional autoregressive models (Besag, 1974) (see Section B of the Supplementary material available at *Biostatistics* online, for details about the implementation of our model).

To fit the model in R-INLA we first define the likelihood (Poisson in our proposal as we model count data). The second stage is to define the latent Gaussian fields, which in our case are given by $({\boldsymbol{\alpha}}',{{\boldsymbol{\Psi}}}',{{\boldsymbol{\Gamma}}}',{\boldsymbol{\Delta}}')'$. And the third stage is to set the hyperparameters that control the precision matrices in the latent fields. In our model the set of hyperparameters are the elements of ${{\boldsymbol{\Sigma}}}_{\psi}$, ${{\boldsymbol{\Sigma}}}_{\gamma}$, $\lambda_1$, and the precision parameters for the interaction terms $(\tau_1,\ldots,\tau_J)$. 

The choice of prior distributions for the hyperparameters is an important issue in Bayesian statistics and some ideas are discussed here. Regarding the covariance matrices of the coefficients of the spatial and temporal P-splines for the different crimes, a Wishart distribution is considered to obtain valid covariance structures (Kuismin and Sillanpää, 2016). In particular, ${\boldsymbol{\Sigma}}_{\psi}\sim {\rm Wishart}(\upsilon,\sigma^2_{\psi}\mathbf{I}_J)$, and ${\boldsymbol{\Sigma}}_{\gamma}\sim {\rm Wishart}(\upsilon,\sigma^2_{\gamma}\mathbf{I}_J)$, where $J$ indicates the number of crimes, $\upsilon$ represents the degrees of freedom, and $\sigma^2_{\psi}$ and $\sigma^2_{\gamma}$ are fixed values. The prior mean is $\upsilon \sigma^2\mathbf{I}_J$ and the greater the value of $\sigma^2$, the less informative the prior is (see Chung *and others,* 2015, for a justification of this prior). The choice of priors on the covariance matrices is a challenge because some parameters are not identifiable and we need to set the degrees of freedom equal to $\upsilon=2J+1$ to make the prior more informative (see Palmí-Perales *and others,* 2021). Regarding the smoothing/precision parameters $\lambda$’s in the precision matrices of the P-spline coefficients, a uniform distribution in the interval $(0,100)$ is considered for $1/\sqrt{\lambda_1}$. As it has been said previously, the parameters $\lambda_2$ and $\lambda_3$ are set equal to one as they are subsumed in the matrices ${{\boldsymbol{\Sigma}}}_{\psi}^{-1}$ and ${{\boldsymbol{\Sigma}}}_{\gamma}^{-1}$ respectively. Also improper uniform distributions in $(0,\infty)$ are considered for the standard deviations $\sigma_{\delta_j}=1/\sqrt{\tau_j}$ of the spatio-temporal interaction (Ugarte *and others,* 2017). As pointed out by one reviewer, we do not have a formal proof about the posterior propriety of our model. Here we follow the ideas in Gelman (2006), where a noninformative uniform prior density on standard deviation parameters is recommended. Finally, we would like to remark that details on how to implement these prior distributions in R-INLA through our multivariate “$\textsf{rgeneric}$” construction can be found in Section B of the Supplementary material available at *Biostatistics* online.

Our models incorporate crime-specific intercepts, P-splines for space, P-splines for time, and spatio-temporal interactions of the type proposed by Knorr-Held (2000). As the crime-specific spatial surfaces (spatial P-splines) and the crime-specific temporal trends (temporal P-splines) also include an intercept, identifiability issues arise, and constraints are necessary. All identifiability constraints would yield to the same functions up to additive constants. A possibility is to put sum to zero constraints on the coefficients

$$
\begin{equation*}
\sum_{m=1}^{k_1k_2}\psi^{(j)}_{m} = 0, \quad \sum_{m=1}^{k_3}\gamma^{(j)}_{m} = 0,\quad\forall j=1,\ldots,J. \\
\end{equation*}$$

As pointed out by one reviewer, these sum to zero constraints seem similar to those used in Markov random field models. Though there are some connections between Markov random fields and splines (see Belitz and Lang, 2008), the reason why these constraints look similar is because the coefficients of the P-splines are modeled as Markov random fields. In particular, the coefficients of the temporal P-splines are modelled with random walks of first or second order. Similarly, the prior for the coefficients of the spatial P-splines combine random walks for longitude and latitude. Though these constraints identify the model, according to Wood *and others* (2013) linear dependence with the intercept leads to excessively wide credible intervals, and it is recommended to make the smooth functions orthogonal to the intercept, that is, to center the smooth functions

$$
\begin{equation*}
\sum_{i=1}^{I}f_{j}(x_{1i},x_{2i}) = 0, \quad \sum_{t=1}^{T} f_{j}(x_{3t})= 0,\quad\forall j=1,\ldots,J. \\
\end{equation*}$$

Also, the interaction effects overlap with the main spatial and temporal P-spline terms and additional constraints are required (see Goicoa *and others,* 2018). In particular, for the Type II interaction, the one selected for our real data, the required constraints are $\sum_{t=1}^{T}\delta_{itj} = 0,\,\, \forall i=i,\ldots,I,\,\,\forall j=1,\ldots,J$ if we consider the precision matrix of a first order random walk. Additionally, the $\lambda_3$ parameter in the precision matrix of the temporal P-splines is not identifiable as it can be subsumed in the matrix ${\boldsymbol{\Sigma}}_{\gamma}^{-1}$ (or $\sigma^2_t=1/\lambda_3$ is subsumed in ${\boldsymbol{\Sigma}}_{\gamma}$). Similarly, the parameters $\sigma_{\gamma j}$, $j=1,\ldots,J$ are not identifiable, so inferences on these quantities should be precluded. Though we have set the parameter $\lambda_3$ equal to one, this is arbitrary and the parameters cannot be interpreted as standard deviations of the P-spline coefficients. On the contrary, the correlation parameters $\rho_{\gamma jk}$ are identifiable and can be perfectly interpreted (see Section C of the Supplementary material available at *Biostatistics* online). The same comments apply to the $\lambda_2$ parameter in the precision matrix $\mathbf{P}_s$ of the spatial P-splines. Though it is set equal to one, the parameters $\sigma_{\psi j}$, $j=1,\ldots,J$ loose their meaning of standard deviations. However, the correlation parameters $\rho_{\psi jk}$ are identifiable.

## 4. Case study: rape, assault, cruelty by husband and relatives, and kidnapping and abduction in Maharashtra, India

In this section, data on crimes against women in the Indian state of Maharashtra over the period 2001–2013 are analyzed using the methodology developed in the previous sections. Data consist of the reported number of cases in each of the 34 districts of Maharashtra for four crimes described in the Indian Penal Code (IPC) (Government of India. Legislative Department, 1860). Namely, *rape*, *assault or criminal force to woman with intent to outrage her modesty*, *cruelty by husband or relatives of husband*, and *kidnapping and abduction*. In the rest of the article, we will refer to these crimes by the shorter names rape, assault, cruelty, and kidnapping. A succinct description of these crimes is provided in Section D of the Supplementary material available at *Biostatistics* online. For a complete definition of the crimes, the reader is referred to the IPC.

### 4.1. Descriptive analysis

**Fig. 1.**
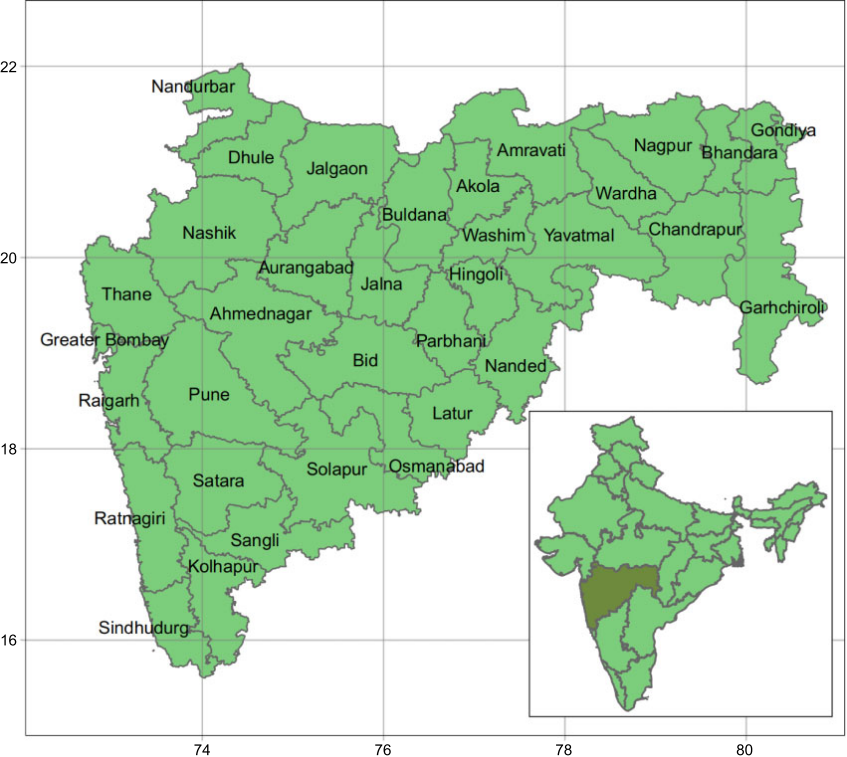
Map of the administrative division of Maharashtra into districts and its location in the west middle of India (bottom right corner).

Maharashtra is located in the middle west of the Indian peninsula, see Figure 1, and according to the 2011 census (see https://www.census2011.co.in), it is the second most highly populated state in the country (112 374 333 inhabitants), surpassed only by Uttar Pradesh. It is also the third largest state of India with a total of 307 713 km$^2$. The overall literacy rate is 82.34%, about 8 percentage points over the overall literacy rate in India (74.04%). Similar to all Indian states, male literacy rate (88.38%) is greater than female literacy rate (75.87%), though these figures are well above the Indian male (82.14%) and female (65.46%) literacy rates. Sex ratio (number of females per 1000 males) is 922, slightly lower than the sex ratio in whole India (933). The majority religion (79.83%) is Hindu.

In recent years, the number of crimes against women in Maharashtra has grown tremendously. According to National Crime Record Bureau, NCRB, (https://ncrb.gov.in/crime-in-india), in the study period the reported incidence of crimes against women has doubled, from 12 524 in 2001 to 24 895 in 2013. In terms of rates, the increase is even worse as the overall rate of crimes against women in 2013 (44.9) is about 3.5 higher than in 2001 (12.9).

**Fig. 2.**
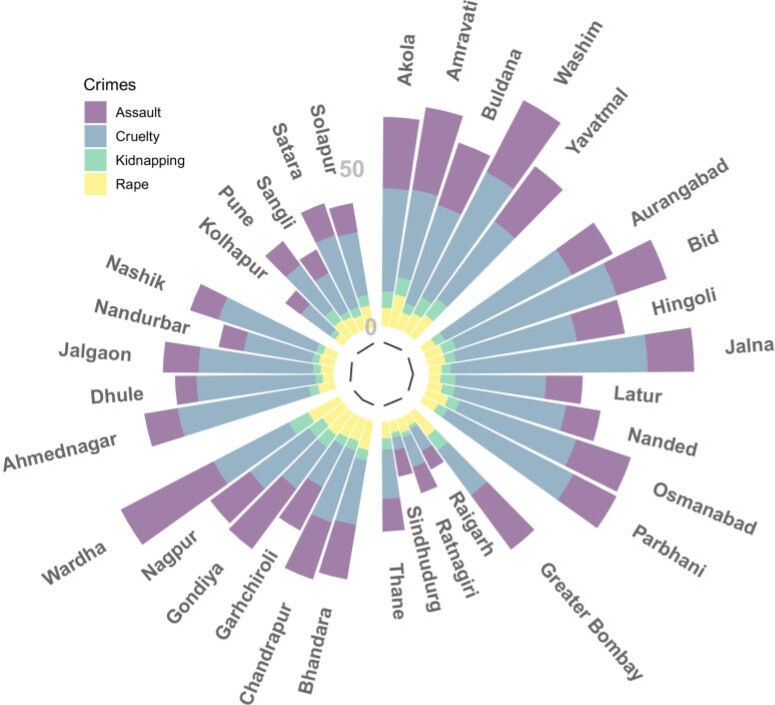
Incidence rates of crimes against women in Maharashtra between 2001 and 2013.

One of the most endemic form of violence against women in different countries of the world is the abuse of women by their intimate partner. The problem is even more serious as it has one of the highest rates of underreporting (see Visaria, 1999; Heise *and others,* 1994, and the references therein). This is also the case of India, where rapes and assaults are also underreported because of shame or fear to incomprehension and social rejection (Koss, 1992). Unfortunately, the state of Maharashtra is not an exception. Figure 2 displays a circular barplot with the crude rates (per 100000 women between the age of 15 and 49 years) of the four different crimes considered in this paper by district. Clearly, cruelty by husband or relatives is the crime against women with the highest incidence, followed by assault. During the 13 years of the study period, a total of 21 049 rapes, 47 351 assaults, 12 727 kidnappings, and 88 905 cases of cruelty were committed in this state. Interestingly, differences are found among the districts. In general, the lowest rates of crimes correspond to the most western districts with the exception of Greater Bombay, whereas districts in the central and northeastern part of the state have the highest rates of crimes. Some descriptive statistics regarding the number of cases of the four crimes in the first and last year of the study period are shown in Table 1.

**Table 1.** Minimum (min), first quartile ($q_{.25}$), mean, third quartile ($q_{.75}$), maximum (max), standard deviation (sd), and coefficient of variation (cv) of the number of crimes against women in the districts of Maharashtra in 2001 and 2013.

Crime	Year	min	Mean	$q_{.25}$	Median	$q_{.75}$	max	sd	cv
Rape	2001	7	25.50	16.75	38.29	43.75	146	33.15	1.30
	2013	11	58.00	40.00	90.09	101.00	466	96.51	1.66
Assault	2001	17	72.50	50.50	83.03	101.00	302	56.39	0.78
	2013	44	166.00	99.50	239.18	283.50	1342	247.74	1.49
Cruelty	2001	8	148.50	90.75	179.12	217.75	496	119.58	0.81
	2013	12	202.50	117.25	251.24	373.50	741	187.74	0.93
Kidnapping	2001	1	13.50	7.00	17.97	21.50	67	15.71	1.16
	2013	5	37.50	21.00	55.12	52.00	292	64.66	1.72

**Fig. 3.**
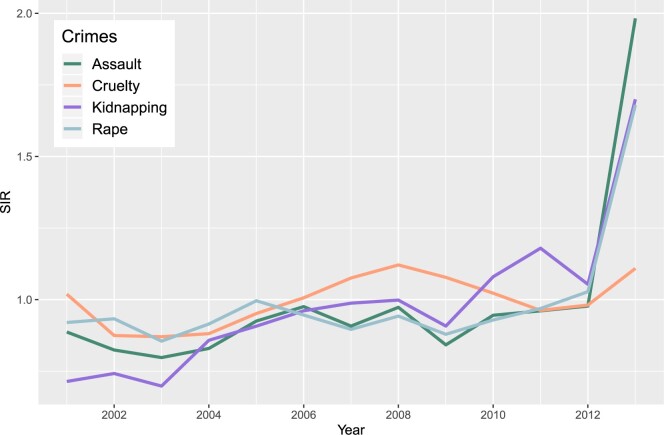
Standardized incidence rate (SIR) by crime.

Figure 3 displays the evolution of the standardized incidence ratio (SIR) for the four crimes. A SIR over one indicates that the number of cases for one crime in a particular year is greater than expected in comparison with the whole study period. A close inspection of this figure reveals rather flat trends for rape, assault, and cruelty until 2012. Then, a strong rise is observed in 2013 for rape and assault. Regarding kidnapping, a steady growth in SIR is observed along the period, with a pronounced increase in the last year. According to some authors, this increase may be attributed to an improvement in the victim support system (Raj and McDougal, 2014).

To identify any potential relationship between the crimes, we compute Pearson correlations between the overall spatial patterns of each crime. That is, we compute the SIR for each district and crime in the study period and calculate the corresponding correlations. The greatest correlations were found between rape and assault (0.79), rape and kidnapping (0.72), and assault and kidnapping (0.81). The correlation of these three crimes with cruelty are low. Similarly, we compute Pearson correlations between the overall temporal trends shown in Figure 3. Again, the highest correlations are found between rape and assault (0.99), rape and kidnapping (0.87), and assault and kidnapping (0.90). The high correlations between these three crimes may be explained because all of them are related in a greater or less extent to sexual offences. However, the statistical analysis has to confirm these exploratory findings.

### 4.2. Model fitting using INLA

#### 4.2.1. Model fitting and model selection

The multivariate P-spline models proposed in Section 2 are implemented to analyze the incidence of the four crimes in Maharashtra. To overcome identifiability issues, the smooth functions are centered as this proposal provides narrower credible intervals. To fit the models, cubic B-splines with first and second order penalties are used for the spatial and temporal dimensions. For longitude and latitude, 10 equidistant internal knots are considered, and for the temporal dimension, 5 internal knots are chosen (see Ruppert, 2002). The final dimensions of the spatial and temporal B-spline bases are $34\times 144$ and $13\times 7,$ respectively. Previous to fit the multivariate P-spline models, a set of univariate P-spline models for each crime has been run. In particular, different priors combining first- and second-order random walks for the spatial and temporal coefficients have been considered. The four types of interactions defined by Knorr-Held (2000) have also been examined. According to different model selection criteria, the Deviance Information Criterion (DIC) (Spiegelhalter *and others,* 2002) and the Watanabe Akaike Information Criterion (WAIC) (Watanabe, 2010), and the Logarithmic Score (LS) (Gneiting and Raftery, 2007), a measure of model prediction performance, first-order penalties for space and time together with Type II interactions for the spatio-temporal term were the best options for all crimes (results not shown). Consequently, the joint multivariate P-spline model given in (2.4) is fitted considering a Type II spatio-temporal interaction for all crimes. We also examine RW1 and RW2 prior distributions for the coefficients. Different values of ($\sigma^2_{\psi},\sigma^2_{\gamma}$) for the Wishart prior on ${\boldsymbol{\Sigma}}_{\psi}$, and ${\boldsymbol{\Sigma}}_{\gamma}$ are considered: (1,1), (1,10), (10,1), (10,10), and (100,100). The model selection criteria, DIC, WAIC, and LS point towards the combination (1,10) and those are the values considered in this article.

The model accounts for potential relationships between the different crimes through the between-crime correlations among the coefficients of the P-splines (spatial and temporal). Additionally, and given that the temporal trends for some crimes are rather flat, we also consider models without correlation between the coefficients of the temporal P-splines, that is, ${\boldsymbol{\Sigma}}_{\gamma}=\mathrm{diag}(\sigma^2_{\gamma 1}, \ldots, \sigma^2_{\gamma J})$. Table 2 displays model selection criteria for the multivariate P-spline models fitted with different combinations of RW1 and RW2 priors for the coefficients of the spatial and temporal P-splines. According to DIC, WAIC, and LS, the best candidate is a model accounting for correlation between the coefficients of the spatial and temporal B-splines, and a RW1 prior distribution for the coefficients. This is the model we finally select to analyze the four crimes.

**Table 2.** Model selection criteria, DIC, WAIC, and LS, for multivariate models.

	Temporal	Order pen.						
${\rm vec}({\boldsymbol{\Delta}})$				$\overline{D}$	$p_D$	DIC	WAIC	LS
	correlations	Spat.	Temp.					
		RW1	RW1	12199.636	877.445	**13077**.**081**	**13089**.**419**	**3**.**880**
			RW2	12567.139	685.262	13252.401	13455.632	3.943
	TRUE							
		RW2	RW1	12205.419	883.941	13089.360	13102.664	3.886
			RW2	12565.742	685.651	13251.394	13453.671	3.942
Type II								
		RW1	RW1	12203.965	885.839	13089.804	13104.674	3.888
			RW2	12568.206	695.589	13263.795	13470.460	3.951
	FALSE							
		RW2	RW1	12206.555	895.889	13102.444	13116.795	3.894
			RW2	12567.505	696.836	13264.341	13471.199	3.950

In general, posterior medians of the quantities of interest (relative risks, spatial, temporal, and spatio-temporal patterns) are rather similar to those obtained using univariate models. However, estimates are more precise with the multivariate proposals.

We have computed 95% credible intervals for the relative risks, the spatial pattern, the temporal trends, and the spatio-temporal interaction term for each of the four crimes analyzed in Maharashtra. On average, the selected multivariate P-spline model provides narrower credible intervals for all quantities of interest. The most remarkable point here is that the constraints affect the credible intervals. In particular, using sum to zero constraints on the coefficients of the smooth functions leads to much wider credible intervals than centering the functions.

Computations were run on a twin superserver with four processors, Intel Xeon 6C and 96GB RAM, using the R-INLA version 21.02.23. All models in the article were fitted using the simplified Laplace strategy in INLA, which provides a good compromise between computing time and accuracy. Models were also fitted using the Gaussian strategy in INLA, which is much faster than the simplified Laplace strategy, though less precise. Some small differences were found in model selection criteria, but the same model was selected and results were pretty similar.

#### 4.2.2. Joint analysis of four crimes against women in Maharashtra

In this subsection, the spatio-temporal patterns of the four crimes in the state of Maharashtra are examined using the selected multivariate P-spline model.

**Fig. 4.**
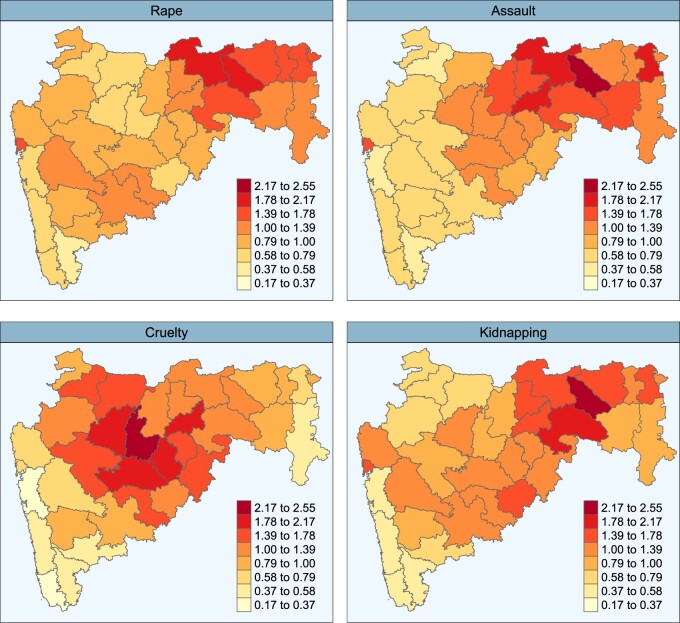
Posterior median of the district-specific spatial risk for rape (top left), assault (top right), cruelty (bottom left), and kidnapping (bottom right).

The underlying spatial patterns and the global temporal trends may be very informative as similarities between crimes could be detected. The district-specific spatial risk for each crime, $\exp(f_{j}(x_{1i},x_{2i}))$, is related to the idiosyncrasy of the districts. It captures the risk associated to a spatial location, and it may reflect the effect of potential spatial risk factors such as certain traditions or demographic and socio-economic characteristics specific to certain districts or regions in the state of Maharashtra. Posterior medians of the district-specific spatial risk for each crime are displayed in Figure 4. The maps with the exceedance probability, that is, $P(\exp(f_{j}(x_{1i},x_{2i}))>1|\mathbf{O})$ are shown in Figure E.1 in Section E of the Supplementary material available at *Biostatistics* online. A Northeast–Southwest gradient (following the largest diagonal axis of the map) can be observed very clearly for rape, assault, and kidnapping, the pattern being smoother for kidnapping. Though some differences exists, the spatial patterns of these three crimes are rather similar. On the other hand, the spatial pattern of cruelty by husband and relatives is different. For this crime, districts with high risk are mainly located in the central part of the map, and a Northwest–Southeast gradient can be envisaged.

The estimated posterior medians of the correlations between the coefficients of the spatial P-splines confirm these findings. Table 3 displays these posterior correlations below the main diagonal. Significant correlations are highlighted in bold. The posterior correlation between rape and assault is particularly high with a posterior median of 0.824. This may point towards underlying spatial factors affecting both crimes. The posterior correlations between rape and kidnapping, and between assault and kidnapping are weaker though significant, with estimated posterior medians 0.433 and 0.474, respectively. The spatial pattern for cruelty is different with most of the high risk districts mainly located in the central part of the map. In fact, as we move away from the center, the map is becoming increasingly lighter. The posterior correlations between the coefficients of the spatial P-splines of cruelty and assaults is 0.267, but it is on the verge of loosing the statistical significance. The posterior correlations between the coefficients of the spatial P-splines of cruelty and the rest of crimes are not significant.

**Table 3.** Estimated correlations (posterior medians and 95% credible intervals) between the spatial P-spline coefficients (below main diagonal) and between the temporal -spline coefficients (above main diagonal). Significant correlations are highlighted in bold.

Crime	Rapes	Assaults	Cruelty	Kidnapping
Rapes	$\ddots$	**0.766**	**0.191**	**0.448**
		(0.701, 0.791)	(0.171, 0.207)	(0.419, 0.465)
Assaults	**0.824**	$\ddots$	**0.493**	**0.676**
	(0.677, 0.902)		(0.477, 0.518)	(0.655, 0.688)
Cruelty	0.053	**0.267**	$\ddots$	**0.224**
	($-$0.192, 0.251)	(0.018, 0.446)		(0.186, 0.245)
Kidnapping	**0.433**	**0.474**	0.257	$\ddots$
	(0.158, 0.603)	(0.220, 0.625)	($-$0.029, 0.465)	

**Fig. 5.**
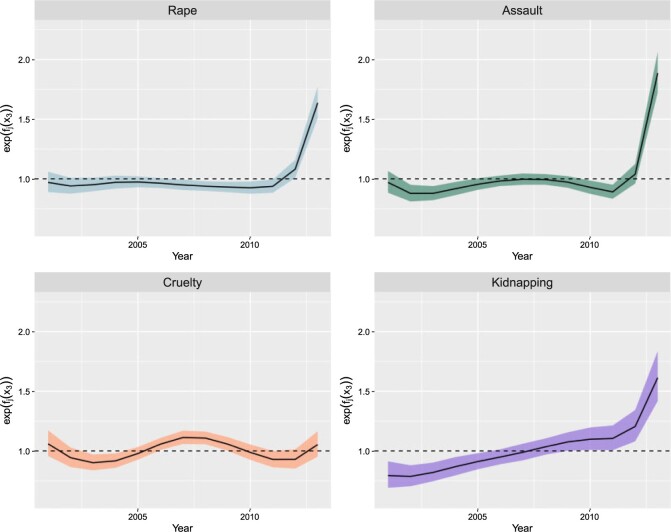
Temporal pattern of incidence risks for rape, assault, cruelty, and kidnapping.

The global temporal evolution of each crime in Maharashtra is revealed by the crime-specific temporal component, $\exp(f_{j}(x_{3t}))$, and may reflect if time-referenced events, such as certain policies, changes in Government, or general social transformations affect the incidence of the crimes in different ways. The posterior medians of $\exp{(f_{j}(x_{3t}))}$ for each crime and the 95% credible intervals are displayed in Figure 5. Trends for rape and assault are fairly flat from 2001 to 2012 with a marked growth in the last year. The temporal trend for cruelty is also rather flat, but it shows a wave-shape, something that is not observed in rape and assault. An slight upturn is observed at the end of the period. A different behavior is observed for kidnapping, which shows a steady growth throughout the period.

The posterior correlation between the coefficients of the temporal P-splines are displayed above the main diagonal in Table 3. Significant correlations are highlighted in bold. The posterior correlation between the coefficients of the temporal P-splines for rape and assault is high (0.766). Lower correlations are observed between rape and cruelty (0.191) and between cruelty and assault (0.493). Regarding kidnapping, the posterior correlation with assault is moderate-high (0.676), and it is moderate or mild with rape (0.448) and cruelty (0.224).

The interaction term $\delta_{itj}$ allows a different time evolution for each area and disease. Although the same type of interaction (Type II) is considered for the four crimes, different precision/variance parameters are allowed for each crime. More precisely, the posterior medians of the standard deviations with a 95% credible interval are 0.110 (0.099, 0.128) for rape, 0.133 (0.117, 0.151) for assault, 0.150 (0.136, 0.165) for cruelty, and 0.184 (0.167, 0.209) for kidnapping. This indicates a different amount of smoothing for each crime. Area-specific temporal trends, that is, the posterior medians of $\exp(\delta_{itj})$, (with 95% credible intervals) are shown in Figure E.2 in Section E of the Supplementary material available at *Biostatistics* online for three districts located in different areas of Maharashtra: Aurangabad (central part of the state), Garhchiroli (in the northeast corner), and Greater Bombay (in the middle western coast). The specific temporal evolution in each area is clearly different indicating that in some districts (Greater Bombay) this trend increases, whereas in other districts (Aurangabad and Garhchiroli) the area-specific temporal trend decreases or it is flat.

To save space, the evolution of the geographical distribution of the relative risk is provided in Section E of the Supplementary material available at *Biostatistics* online. Figures E.3, E.4, E.5, and E.6 of the Supplementary material available at *Biostatistics* online show the posterior medians of the relative risks, $R_{itj}$, (top) and posterior probabilities of risk exceedance, $P(R_{itj}>1|{\bf O})$ (bottom) in the study period, for rape, assault, cruelty, and kidnapping, respectively. In general, the risk distribution for rape, assault, and cruelty remains stable during the period with an increase in the last year. The pattern for kidnapping is different with a steady increase of the risk along the period. In summary, these risk patterns confirm the Northeast–Southwest gradient for rape, assault, and kidnapping, with most of the high risk areas in the northeast of the state, and a Northwest–Southeast gradient for cruelty with most of the high-risk areas concentrated in the central part of the map.

**Fig. 6.**
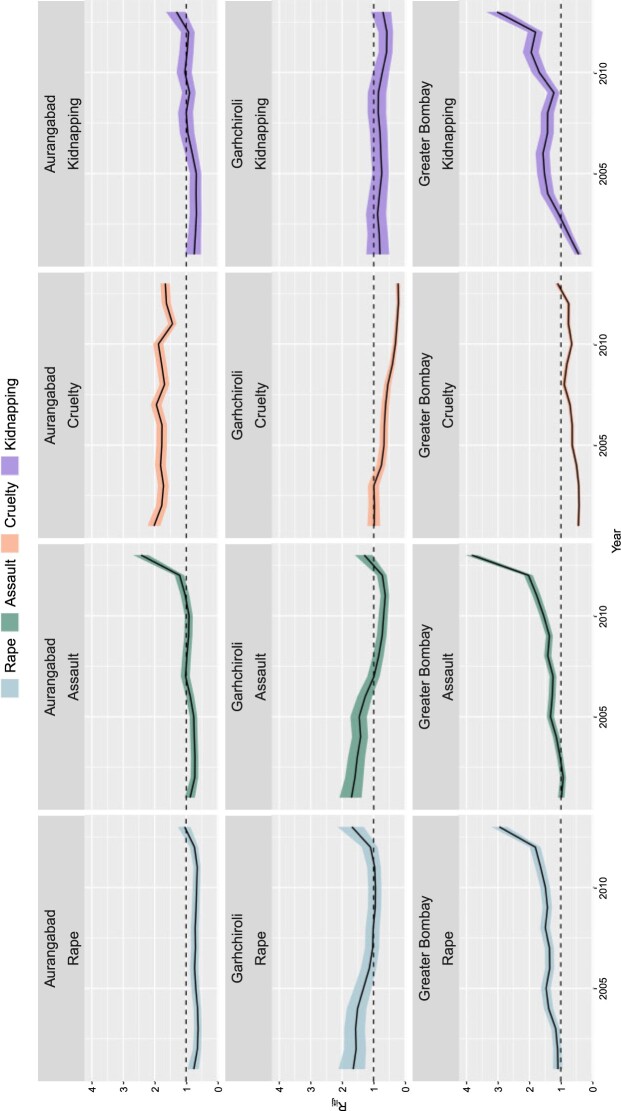
Relative risk evolution (posterior median of $R_{itj}$) for three selected districts: Aurangabad, Garhchiroli, and Greater Bombay.

Figure 6 displays the relative risk evolution of the four crimes in Aurangabad, Garhchiroli, and Greater Bombay. The relative risk of cruelty in Araungabad remains nearly constant and is about twice the risk of whole Maharashtra. Garhchiroli has low relative risks for all crimes, though an upturn is observed for rape and assault at the end of the period. Finally, Greater Bombay shows increasing and significantly high risk for rape, assault, and kidnapping. The risk for cruelty remains low during the study period.

**Fig. 7.**
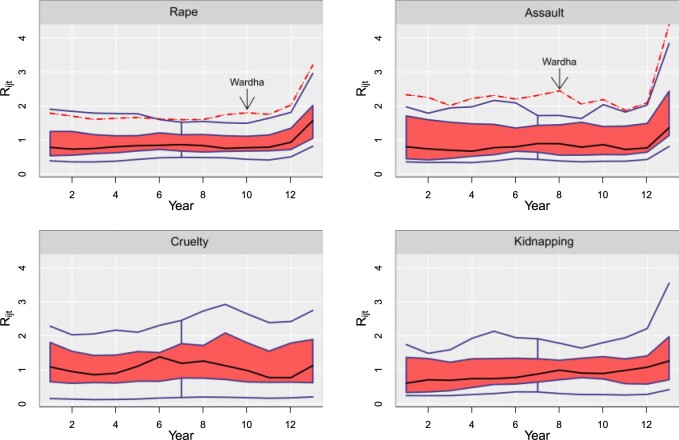
Functional boxplots of the final relative risk trends for rape (top left), assault (top right), cruelty (bottom left), and kidnapping (bottom right).

To conclude the analysis, it would be helpful to identify outlying districts regarding their relative risk temporal evolution. Sun and Genton (2011) propose a functional boxplot to visualize functional data and to detect outlying functions. Figure 7 displays the functional boxplots of the final relative risk trends for rape (top left), assault (top right), cruelty (bottom left), and kidnapping (bottom right). According to the functional boxplot, the district of Wardha, in the northeastern corner of Maharashtra, is an outlying district with regard to rape and assault, with a risk greater than the rest of districts. Interestingly, any outlying district is found in relation to cruelty and kidnapping. It remains unknown why this particular district is an outlier regarding rape and assault, and it is a matter of investigation for social researchers and anthropologists. Some infrastructure indicators (Directorate of Economics & Statistics, 2017) in the period 2013–2014 (2013 is the last year of our study period) reveal that Wardha was one of the districts with the lowest number of total road kilometers, and also with less post offices. This could indicate some form of isolation that could favor a sense of impunity about sexual crimes. However, this is mere speculation and further insight into this district is needed.

A natural question about our proposed model is the sensitivity of the results to the number of knots and their location in space and time. For low rank splines without penalties the number and location of knots are very important (see Rice and Wu, 2001), but in penalized splines the penalty relaxes the relevance of the number and location of knots. We have run the analysis changing the number of knots in space (longitude and latitude) and in time to assess how sensitive the results are with respect to these choices. Increasing the number of knots in longitude and latitude beyond 10 increases computing time, does not reduce model selection criteria, and the estimated spatial patterns do not change. On the other hand, using more than five internal knots in time seems to overfit the temporal trends as little smoothing is observed. Regarding the posterior correlations, some sensitivity has been observed. If the number of internal knots decreases, some correlations between spatial patterns become nonsignificant. This is probably due to an oversmoothing. Something similar happens when we modify the number of knots in time. In general, the correlation between the spatial patterns do not change too much, but some differences are found in the posterior correlations between the temporal trends.

To conclude this section, and as suggested by one reviewer, we would like to compare our model with other multivariate approaches in the literature. There are interesting proposals that could be explored. Bradley *and others* (2018) propose a multivariate spatio-temporal model based on multivariate Gamma distributions that conjugate with Poisson distributions oriented to high dimensional data. This approach simplifies sampling for use with MCMC. A recent multivariate proposal for spatial disease mapping based on directed acyclic graphs (DAGAR) (see Datta *and others,* 2019) has been proposed by Gao *and others* (2021). Though this proposal depends on the order in which the different responses are modeled, the authors propose Bayesian averaging to overcome this order dependency. Here, we compare our P-spline approach with the M-models proposed by Botella-Rocamora *and others* (2015) in a spatial setting and extended to the spatio-temporal framework by Vicente *and others* (2020b). Both approaches are different as the M-model includes spatial random effects with CAR priors and temporal random effects with first or second order random walk priors. In particular, we consider intrinsic conditional autoregressive priors (ICAR) for space, random walks of first order for time, and a Type II interaction for the spatio-temporal interaction term. The DIC, WAIC, and LS are 13 024.642, 13 013.492, and 3. 847 respectively, which are smaller than those obtained with the P-spline models, but the decrease is about 0.5% in DIC and WAIC and about 0.8% in LS. In general, the M-models based on ICAR priors produce less smoothing than the P-spline models and hence the reduction in the model selection criteria. For the case study considered here, we obtain pretty similar results with both procedures in terms of relative risks estimates and spatial patterns. The main difference is that as our multivariate P-spline model produces smoother temporal trends than the M-model (spatial patterns and temporal trends obtained with the M-model are provided in Figures E.7 and E.8 respectively in Section E of the Supplementary material available at *Biostatistics* online). One advantage of our procedure is that the between-crimes covariance matrix is parameterized in terms of the correlation parameters, so the posterior distributions of these parameters are directly obtained. On the contrary, the M-models are based on the matrix ${\mathbf M}$ which depends on $J\times J$ parameters. This leads to an overparameterization as the covariance matrix only requires $J(J+1)/2$ parameters. This in turn produces less precise estimates of the correlation parameters. Table 4 displays the posterior correlations between the spatial (lower diagonal) and between the temporal (upper diagonal) patterns obtained with the M-model. Comparing these correlations with those in Table 3, we observe rather similar posterior medians for the spatial correlations, though the credible intervals are wider with the M-model. The differences are more pronounced in the correlations between the temporal patterns. The M-model leads to rather wide credible intervals for most of the correlations. Finally, some differences are expected as the P-spline approach provides correlations between the coefficients, whereas the M-model gives correlation between ICAR spatial random effects and temporal random effects modeled with random walk priors.

**Table 4.** Estimated correlations (posterior medians and 95% credible intervals) between spatial patterns (below main diagonal) and between the temporal trends (above main diagonal) obtained with the M-model. Significant correlations are highlighted in bold.

Crime	Rapes	Assaults	Cruelty	Kidnapping
Rapes	$\ddots$	**0.976**	0.509	**0.817**
		(0.840, 0.995)	($-$0.158, 0.846)	(0.421, 0.956)
Assaults	**0.659**	$\ddots$	0.610	**0.823**
	(0.400, 0.820)		($-$0.031, 0.889)	(0.453, 0.958)
Cruelty	0.044	**0.334**	$\ddots$	0.326
	($-$0.302, 0.361)	(0.017, 0.601)		($-$0.396, 0.782)
Kidnapping	**0.558**	**0.557**	0.286	$\ddots$
	(0.275, 0.758)	(0.280, 0.743)	($-$0.053, 0.554)	

## 5. Discussion

Violence against women is a social problem all over the world, though in some nations is exacerbated. India is one of such countries where this problem is in the spotlight because of the large number of affected women and the cruelty of some forms of crimes. However, even society in general is getting more and more concerned about this issue, factors that may be related to crimes against women are still unclear and they can be very different depending on the region. India is a paradigmatic case as the wide diversity of traditions, religious beliefs, and social practices hamper the identification of such factors. In this context, statistical methodology in general, and spatio-temporal models for areal count data in particular may help to understand this intricate phenomenon. In the problem considered here this is crucial given that we could not obtain spatio-temporal socio-demographic covariates at district level.

In this article, we present new methodology to model different crimes simultaneously. This is a clear benefit over univariate analyses as it is possible to establish associations between different forms of crimes that may help to understand connections between them, and hence to better comprehend the problem. In particular, multivariate spatio-temporal models based on P-splines are used to analyze jointly four crimes against women in the Indian state of Maharashtra. More precisely, we propose two-dimensional spatial P-splines and one-dimensional temporal P-splines to model the geographical patterns and the temporal trends respectively. Correlations among the coefficients of the P-splines for the different crimes are introduced to look into spatial and temporal associations between the crimes that could point towards connections between them. This joint modeling allows borrowing information from neighboring areas, time points, and also from other crimes, improving the precision of the estimates.

The models have been fitted using integrated nested Laplace approximations in $\textsf{R-INLA}$ and have been built using the “$\textsf{rgeneric}$” construction. As far as we know, this is the first attempt to fit multivariate P-spline models with $\textsf{R-INLA}$. Some comments regarding the model complexity are important. First, the smoothing parameter of the temporal P-spline and one of the smoothing parameters of the spatial P-spline are subsumed in the covariance matrix between the coefficients of the temporal and spatial P-splines, respectively. Hence the variance parameters of these matrices are not identifiable. We have set those smoothing parameters equal to one, but this is arbitrary and the variance parameter are no longer interpretable. Consequently inference about the nonidentifiable parameters is avoided. However, the more relevant correlation parameters are identifiable. Second, the spatial and temporal effects include an intercept, and identifiability constraints are needed. Though the relative risk estimates are invariant to the constraints, the credible interval for the smooth functions can be greatly affected. In particular, sum to zero constraints on the coefficients lead to much wider credible intervals than forcing orthogonality between the intercept and the functions (centering the functions). This is particularly noticeable for the temporal P-splines. Consequently, in the real data analysis, the smooth functions have been centered. Additionally, the interaction terms are centered within each area.

To enrich the article, we have compared our multivariate P-spline model with other existing proposals in the literature. In particular, with the M-model based approach developed by Botella-Rocamora *and others* (2015). Both proposals are implemented in R-INLA and provide similar results. Though the M-models present slightly lower values of the different model selection criteria, the multivariate P-spline model presents some advantages. The first one is that the between-crime covariance matrices are parameterized in terms of the correlation parameters and the posterior distributions of these quantities are directly obtained with R-INLA, unlike the M-models in which the correlation parameters have to be obtained by a resampling procedure. Second, the M-model approach is overparameterized as it considers $J\times J$ parameters to obtain the between-crime covariance matrices when only $J\times(J+1)/2$ parameters are required. This leads to wider credible intervals for the correlation parameters and consequently, less precise estimates. Additionally, in Adin *and others* (2017), P-spline models show a good performance in terms of sensitivity (detecting true high risk areas) and specificity (discarding false positives), though we acknowledges that this issue requires further research in a multivariate framework.

A potential limitation of our model (and also of the M-model approach) is that the number of parameters in the between-crimes covariance matrices increases considerably with the number of crimes. For example, adding one more crime to our study leads to 15 parameters in the covariance matrices instead of 10. This is important as increasing the number of variance parameters may raise computing time in INLA. A solution to reduce dimension could be factors models, but this has to be explored. Regarding augmenting the number of areas and time points, we are studying a new strategy based on making groups of small areas and fitting the models in those smaller groups of regions (see Orozco-Acosta *and others,* 2021). This strategy has proven effective with univariate CAR models, but has to be further studied in multivariate settings and with P-spline models. Finally, as pointed out by one reviewer, INLA relies on numerical approximations instead of sampling, and trace plots to check convergence of the posterior distributions are not available. Model checking is particularly relevant with models such as the one proposed in this paper with a high number of hyperparameters whose distributions are not normal. In general, convergence issues can be detected in INLA when unreasonable large posterior standard deviations of the parameters of interests are obtained, and if the posterior marginal densities of the hyperparameters present spikes (see Martins *and others,* 2013, for the algorithm used in INLA for the posterior distribution of the hyperparameters). In these cases useless credible intervals (too wide) are obtained. In our experience, if similar results are obtained using the Gaussian and the simplified Laplace approximations, the posteriors are well approximated. In any case, we strongly recommend a careful inspection of the results to detect the aforementioned issues that indicate bad approximations of the posterior distributions of the quantities of interest. The last concern regarding our model that we would like to comment on is the prior distributions on the covariance matrix. Here, we choose Wishart priors on the covariance matrices (Chung *and others,* 2015). Given that some parameters of these matrices are not identifiable, we need to increase the degrees of freedom of the Wishart distribution to make the prior more informative and explore different values for the parameters $\sigma^2_{\psi}$ and $\sigma^2_{\gamma}$.

Regarding the real case study in Maharashtra, results are very interesting. The analysis reveals similarities between the spatial patterns of rape, assault and kidnapping with a Northeast–Southwest gradient, whereas the spatial pattern for cruelty is different and a Northwest–Southeast gradient is observed. With respect to temporal evolutions the greatest similarities are those of rape and assault. Our study also identifies districts with high risk for some or all crimes examined here. Moreover, functional boxplots discover Wardha as an outlying district with a risk of rape and assault greater than the rest of districts. We firmly believe that our findings will be useful for social researchers and anthropologists to disentangle the complex phenomenon of violence on women. Additional research in those districts could bring light to identify potential risk factors that may be related with the crimes. So far, we could only make hypotheses based on our results and existing literature. For example, it has been documented that in urban slums in Bombay, the risk of cruelty increases if the man in the household consumes alcohol (see Begum *and others,* 2015). Other descriptive studies in rural villages (see Jain *and others,* 2004) points towards the predominant role of men over women, economic stress, many people living in one room, or complaints of the mother in law as reasons for the abuse. Collecting information about these characteristics at area level would be the first step to assess their validity as potential predictors.

## Supplementary Material

kxab042_Supplementary_Data

## 6. Software

R code, together with the data set and complete documentation is available in the GitHub of our research group (https://github.com/spatialstatisticsupna/Multivariate_spatio_temporal_P_spline).

## References

[B1] Adin, A. , Martínez-Beneito, M. A., Botella-Rocamora, P., Goicoa, T. and Ugarte, M. D. (2017). Smoothing and high risk areas detection in space-time disease mapping: a comparison of P-splines, autoregressive, and moving average models. Stochastic Environmental Research and Risk Assessment 31, 403–415.

[B2] Aitchison, J. and Ho, C. H. (1989). The multivariate Poisson-log normal distribution. Biometrika 76, 643–653.

[B3] Begum, S. , Danta, B., Nair, S. and Prakasam, C. P. (2015). Socio-demographic factors associated with domestic violence in urban slums, Mumbai, Maharashtra, India. Rural and Remote Health 141, 783–788. 10.4103/0971-5916.160701 PMC4525403 26205021

[B4] Belitz, C. and Lang, S. (2008). Simultaneous selection of variables and smoothing parameters in structured additive regression models. Computational Statistics and Data Analysis 53, 61–81.

[B5] Besag, J. (1974). Spatial interaction and the statistical analysis of lattice systems (with discussion). Journal of the Royal Statistical Society: Series B (Statistical Methodology) 36, 192–236.

[B6] Botella-Rocamora, P. , Martinez-Beneito, M. A. and Banerjee, S. (2015). A unifying modeling framework for highly multivariate disease mapping. Statistics in Medicine 34, 1548–1559. 25645551 10.1002/sim.6423

[B7] Bradley, J. R. , Holan, S. H. and Wikle, C. K. (2018). Computationally efficient multivariate spatio-temporal models for high-dimensional count-valued data (with discussion). Bayesian Analysis 13, 253–310.

[B8] Chung, Y. , Gelman, A., Rabe-Hesketh, S., Liu, J. and Dorie, V. (2015). Weakly informative prior for point estimation of covariance matrices in hierarchical models. Journal of Educational and Behavioral Statistics 40, 136–157.

[B9] Datta, A. , Banerjee, S., Hodges, J. S. and Gao, L. (2019). Spatial disease mapping using directed acyclic graph auto-regressive (DAGAR) models. Bayesian Analysis 14, 1221–1244. 33859772 10.1214/19-ba1177 PMC8046356

[B10] Directorate of Economics & Statistics. (2017). Infrastructure statistics of Maharashtra state, 2013-14 and 2014-15. https://mahades.maharashtra.gov.in/files/publication/Infrast_Statistics2013-14_2014-15.pdf.

[B11] Eilers, P. H. C. , Currie, I. D. and Durbán, M. (2006). Fast and compact smoothing on large multidimensional grids. Computational Statistics & Data Analysis 50, 61–76.

[B12] Eilers, P. H. C. and Marx, B. D. (1996). Flexible smoothing with B-splines and penalties. Statistical Science 11, 89–102.

[B13] Gao, L. , Datta, A. and Banerjee, S. (2021). Multivariate Directed Acyclic Graph Auto-Regressive (MDAGAR) models for spatial diseases mapping. UCLA: Biostatistics. Retrieved from https://escholarship.org/uc/item/88c7t942.

[B14] Gelman, A. (2006). Prior distributions for variance parameters in hierarchical models (comment on article by Browne and Draper). Bayesian Analysis 1, 515–534.

[B15] Gneiting, T. and Raftery, A. E. (2007). Strictly proper scoring rules, prediction, and estimation. Journal of the American Statistical Association 102, 359–378.

[B16] Goicoa, T. , Adin, A., Ugarte, M. D. and Hodges, J. S. (2018). In spatio-temporal disease mapping models, identifiability constraints affect PQL and INLA results. Stochastic Environmental Research and Risk Assessment 32, 749–770.

[B17] Goicoa, T. , Etxeberria, J. and Ugarte, M. D. (2016). Splines in disease mapping. In: Lawson, A. B., Banerjee, S., Haining, R. P., and Ugarte, M. D. (editors), Handbook of Spatial Epidemiology. Boca Raton: Chapman and Hall, pp. 225–238.

[B18] Goicoa, T. , Ugarte, M. D., Etxeberria, J. and Militino, A. F. (2012). Comparing CAR and P-spline models in spatial disease mapping. Environmental and Ecological Statistics 19, 573–599.

[B19] Government of India. Legislative Department. (1860). Indian Penal Code. Retrieved December 22, 2020. http://legislative.gov.in/actsofparliamentfromtheyear/indian-penal-code.

[B20] Heise, L. L. , Raikes, A., Watts, C. H. and Zwi, A. B. (1994). Violence against women: a neglected public health issue in less developed countries. Social Science & Medicine 39, 1165–1179. 7801154 10.1016/0277-9536(94)90349-2

[B21] Jain, D. , Sanon, S., Sadowski, L. and Hunter, W. (2004). Violence against women in India: evidence from rural Maharashtra, India. Rural and Remote Health 4, 304. 15887989

[B22] Jin, X. , Banerjee, S. and Carlin, B. P. (2007). Order-free co-regionalized areal data models with application to multiple-disease mapping. Journal of the Royal Statistical Society: Series B (Statistical Methodology) 69, 817–838. 20981244 10.1111/j.1467-9868.2007.00612.x PMC2963450

[B23] Knorr-Held, L. (2000). Bayesian modelling of inseparable space-time variation in disease risk. Statistics in Medicine 19, 2555–2567. 10960871 10.1002/1097-0258(20000915/30)19:17/18<2555::aid-sim587>3.0.co;2-#

[B24] Koss, M. P. (1992). The under detection of rape: methodological choices influence incidence estimates. Journal of Social Issues 48, 61–75.

[B25] Kuismin, M. and Sillanpää, M. J. (2016). Use of wishart prior and simple extensions for sparse precision matrix estimation. PLoS One 11, e0148171. 26828427 10.1371/journal.pone.0148171 PMC4734711

[B26] Lang, S. and Brezger, A. (2004). Bayesian p-splines. Journal of Computational and Graphical Statistics 13, 183–212.

[B27] Lawson, A. , Banerjee, S., Haining, R. and Ugarte, M. D. (editors). (2016). Handbook of Spatial Epidemiology. New York: Chapman and Hall/CRC.

[B28] Lindgren, F. and Rue, H. (2015). Bayesian spatial modelling with R-INLA. Journal of Statistical Software 63, 1–25.

[B29] MacNab, Y. C. (2007). Spline smoothing in Bayesian disease mapping. Environmetrics 18, 727–744. 10.1002/sim.2868 17357989

[B30] MacNab, Y. C. (2018). Some recent work on multivariate Gaussian Markov random fields. Test 27, 497–541.

[B31] MacNab, Y. C. and Gustafson, P. (2007). Regression B-spline smoothing in Bayesian disease mapping: with an application to patient safety surveillance. Statistics in Medicine 26, 4455–4474. 17357989 10.1002/sim.2868

[B32] Martinez-Beneito, M. (2013). A general modelling framework for multivariate disease mapping. Biometrika 100, 539–553.

[B33] Martínez-Beneito, M. A. and Botella-Rocamora, P. (2019). Disease Mapping: From Foundations to Multidimensional Modeling. Boca Raton: CRC Press.

[B34] Martins, T.G. , Simpson, D., Lindgren, F. and Rue, Havard. (2013). Bayesian computing with INLA: new features. Computational Statistics and Data Analysis 67, 68–83.

[B35] Orozco-Acosta, E. , Adin, A and Ugarte, M. D. (2021). Scalable Bayesian modelling for smoothing disease risks in large spatial data sets using INLA. Spatial Statistics 41, 100496.

[B36] Palmí-Perales, F. , Gómez-Rubio, V. and Martínez-Beneito, M. A. (2021). Bayesian multivariate spatial models for lattice data with INLA. Journal of Statistical Software 98, 1–29.

[B37] Raj, A. and McDougal, L. (2014). Sexual violence and rape in India. The Lancet (Correspondence) 383, 865. 10.1016/S0140-6736(14)60435-9 24607092

[B38] Rice, J. A. and Wu, C. O. (2001). Nonparametric mixed effects models for unequally sampled noisy curves. Biometrics 57, 253–259. 11252607 10.1111/j.0006-341x.2001.00253.x

[B39] Rue, H. and Held, L. (2005). Gaussian Markov Random Fields: Theory and Applications. Boca Raton: Chapman & Hall.

[B40] Rue, H. , Martino, S. and Chopin, N. (2009). Approximate Bayesian inference for latent Gaussian models by using integrated nested Laplace approximations. Journal of the Royal Statistical Society: Series B (Statistical Methodology) 71, 319–392.

[B41] Ruppert, D. (2002). Selecting the number of knots for penalized splines. Journal of Computational and Graphical Statistics 11, 735–757.

[B42] Spiegelhalter, D. J. , Best, N. G., Carlin, B. P. and Van Der Linde, A. (2002). Bayesian measures of model complexity and fit. Journal of the Royal Statistical Society: Series B (Statistical Methodology) 64, 583–639.

[B43] Sun, Y. and Genton, M. G. (2011). Functional boxplots. Journal of Computational and Graphical Statistics 20, 316–334.

[B44] Ugarte, M. D. , Adin, A. and Goicoa, T. (2017). One-dimensional, two-dimensional, and three dimensional B-splines to specify space–time interactions in Bayesian disease mapping: model fitting and model identifiability. Spatial Statistics 22, 451–468.

[B45] Ugarte, M. D. , Goicoa, T. and Militino, A. F. (2010). Spatio-temporal modeling of mortality risks using penalized splines. Environmetrics 21, 270–289.

[B46] Vicente, G. , Goicoa, T., Fernández-Rasines, P. and Ugarte, M. D. (2020a). Crime against women in India: unveiling spatial patterns and temporal trends of dowry deaths in the districts of Uttar Pradesh. Journal of the Royal Statistical Society: Series A (Statistics in Society) 183, 655–679.

[B47] Vicente, G. , Goicoa, T. and Ugarte, M. D. (2020b). Bayesian inference in multivariate spatio-temporal areal models using INLA: analysis of gender-based violence in small areas. Stochastic Environmental Research and Risk Assessment 34, 1421–1440.

[B48] Visaria, L. (1999). Violence against women in India: Evidence from rural Gujarat. In: Duvvury Nata (editor). Domestic Violence in India: A Summary Report of Three Studies. Washington, DC: International Center for Research on Women, pp. 9–17.

[B49] Watanabe, S. (2010). Asymptotic equivalence of Bayes cross validation and widely applicable information criterion in singular learning theory. Journal of Machine Learning Research 11, 3571–3594.

[B50] Wood, S. N. , Scheipl, F. and Faraway, J. J. (2013). Straightforward intermediate rank tensor product smoothing in mixed models. Statistics and Computing 23, 341–360.

