# Supplementary material for: Multivariate Bayesian spatio-temporal P-spline models to analyze crimes against women
Source: Biostatistics. 2021 Dec 27;24(3):562–84. doi: 10.1093/biostatistics/kxab042 (PMC10345996; doi:10.1093/biostatistics/kxab042)
Supplement: kxab042_Supplementary_Data [file kxab042_supplementary_data.zip › kxab042_Supplementary_Data/multivariate_P_SPLINES_supplementary_2021_07_02_v3.pdf]

Biostatistics (2021), **0**, 0, *pp.* 1–15
doi:10.1093/biostatistics/multivariate˙P˙SPLINES˙supplementary˙2021˙07˙02˙v3

Multivariate Bayesian spatio-temporal P-spline models to analyse crimes against women. Supplementary materials

GONZALO VICENTE[1], TOMÁS GOICOA [1,2], MARÍA DOLORES UGARTE[1,2*]

[1]*Department of Statistics, Computer Science, and Mathematics, Public University of Navarre, Campus de Arrosadia, 31006 Pamplona, Spain*

[2]*InaMat*[2]*, Public University of Navarre, Spain*

lola@unavarra.es

A. Introduction

This supplementary material contains additional information to better understand the paper entitled "Multivariate Bayesian spatio-temporal P-spline models to analyse crimes against women" and has the following sections. Section B briefly explains how to implement the multivariate P-spline models in R-INLA. It also provides technical details to derive the joint prior $\pi(\boldsymbol{\theta}^*)$ for the internal parameters required in R-INLA to define the hyperparameters and how to implement this prior distribution. Section C gives a proof of the identifiability of the correlation parameters. Section D provides a brief description of the crimes studied in this paper, and finally, additional figures for the case study are shown in Section E.

*To whom correspondence should be addressed.

© The Author 2021. Published by Oxford University Press. All rights reserved. For permissions, please e-mail: journals.permissions@oup.com

B. "rgeneric" construction to implement multivariate spatial P-splines with INLA

In this section we briefly explain how to build an "rmodel" to fit multivariate spatial P-splines with INLA. A similar construction applies for multivariate temporal P-splines. We first have to define the "rmodel" that we label "generic_mps_spat". Through the "inla.rgeneric.define" function, we create an "inla.rgeneric" object that we call "model.s"

```
model.s <- inla.rgeneric.define(generic_mps_spat, ...)
```

Then, we can embed this "inla.rgeneric" object in the usual INLA syntaxis to fit multivariate spatial P-splines

```
formula.inla <- 0 ~ ... + f(idx, model = model.s, ...) + ...
```

To define the multivariate spatial P-spline model "generic_mps_spat", it is necessary to create some functions that provide the graph (a 0/1 representation of the precision matrix), the precision matrix (in our case $\mathbf{\Sigma}_{\psi}^{-1} \otimes \mathbf{P}_s$), and the log priors. For some guidance and more information about how to build an "rgeneric" function, the following line in R, vignette("rgeneric", package="INLA"), provides documentation.

The "rgeneric" function "generic_mps_spat" needs to parameterize the covariance/precision matrix between crimes in terms of some parameters whose support has to be $\mathbb{R}$, and the variances and correlations can be conveniently recovered through appropriate transformations. More precisely, the "generic_mps_spat" function defined for the multivariate setting requires internal generic parameters, which we will call $\boldsymbol{\theta}$. In particular, for the covariance matrix between the set of P-spline coefficients (spatial or temporal), $J(J+1)/2$ parameters are needed, where J is the number of crimes, that is, $\boldsymbol{\theta} = (\theta_1, \ldots, \theta_{J(J+1)/2})$. The first J parameters, $\theta_1, \ldots, \theta_J$, correspond to the log precision (or $\log(1/\sigma_j^2)$) of each crime. The rest of parameters, $\theta_{J+1}, \ldots, \theta_{J(J+1)/2}$, are related to the correlation parameters between crimes through the expression $\theta_j = \log\left((1+\rho_j)/(1-\rho_j)\right)$,

$j = J+1, \ldots, J(J+1)/2$, where here ρ_j refers to the correlation coefficient between two crimes. In more detail, θ_{J+1} refers to correlation between crimes 1 and 2; θ_{J+2} refers to correlation between crimes 1 and 3, and so on. Using this transformation, the support for each of the internal parameters θ_j, $j = J+1, \ldots, J(J+1)/2$ is $\mathbb{R}$. An additional, θ_{λ_1} parameter is needed for the smoothing parameter λ_1. Note that only the ratio λ_1/λ_2 (o λ_2/λ_1) is identifiable, see Section 2, and consequently we set λ_2 to 1 (similarly, in the case of temporal P-splines we set λ_3 to 1). The smoothing parameter play the role of precision parameter, that is $\lambda_1 = 1/\sigma^2_{\lambda_1}$, and then $\sigma_{\lambda_1} = 1/\sqrt{\lambda_1}$ is standard deviation. As we will use uniform prior distributions on the standard deviations, the parameterization is $\theta_{\lambda_1} = \log(1/\sigma^2_{\lambda_1})$.

It is worth noting that as it has been commented previously, the correlation parameters ρ_j $(j = 1, \cdots, J)$ are identifiable and can be perfectly interpreted. Then, to recover the correlation parameters between crimes, we back transform the internal parameters using

$$\rho_j = g(\theta_j) = 2\exp(\theta_j)/(1+\exp(\theta_j)) - 1,\ j = J+1, \ldots, J(J+1)/2.$$

The choice of prior distributions for the hyperparameters is an important issue in Bayesian statistics and has been discussed in Section 3. Here we explain how to implement these priors distribution in R-INLA. To include the priors within the "generic_mps_spat" function, the joint prior for $\boldsymbol{\theta}^* = (\theta_1, \ldots, \theta_{J(J+1)/2}, \theta_{\lambda_1})$ has to be defined. That is

$$\pi(\boldsymbol{\theta}^*) = Wishart(\upsilon, \sigma^2_\psi \mathbf{I}_J) \times \mathrm{Jac}_{\boldsymbol{\theta}} \times Unif(0, 100) \times \mathrm{Jac}_{\theta_{\lambda_1}},$$

where $Wishart(\upsilon, \sigma^2_\psi \mathbf{I}_J)$ is the prior for the covariance matrix, $\mathrm{Jac}_{\boldsymbol{\theta}}$ is the Jacobian for the change of variable from the elements σ^2's and ρ's in $\boldsymbol{\Sigma}_\psi$ to the θ's (parameters whose support must be $\mathbb{R}$). $Unif(0, 100)$ is the prior for σ_{λ_1} and $\mathrm{Jac}_{\theta_{\lambda_1}}$ is the Jacobian of the transformation $\sigma_{\lambda_1} = \exp(-0.5 * \theta_{\lambda_1})$.

The log of this density is included in the log.prior() function within the "generic_mps_spat" function. More details about this density and how to incorporate it in the "generic_mps_spat"

function are included below.

B.1 *Density of* $\pi(\boldsymbol{\theta}^*)$

Here we show how to derive the density for $\pi(\boldsymbol{\theta}^*)$ for the case of $J = 4$ crimes. The generalization to more crimes is similar. It is required that the parameters in $\boldsymbol{\theta}^*$ have support on $\mathbb{R}$, so we have re-parameterized the σ^2's and ρ's elements in $\boldsymbol{\Sigma}_\psi$ and σ_{λ_1} as

$$\sigma_j = g(\theta_j) = \exp(-0.5 * \theta_j),\ j = 1, \ldots, J,$$
$$\rho_j = g(\theta_j) = 2\exp(\theta_j)/(1 + \exp(\theta_j)) - 1,\ j = J+1, \ldots, J(J+1)/2,$$
$$\sigma_{\lambda_1} = \exp(-0.5 * \theta_{\lambda_1}).$$

The between-crimes covariance matrix $\boldsymbol{\Sigma}_\psi$ requires $J(J+1)/2 = 10$ parameters. The first four parameters are the variances σ_j^2, and the 6 remaining parameters are the covariances. Let us denote by $X_j = \sigma_j^2 = e^{-\theta_j}, j = 1, \ldots, 4$, and by

$$X_5 = \rho_{12}\sigma_1\sigma_2 = \left(2e^{\theta_5}/(1 + e^{\theta_5}) - 1\right) e^{-\theta_1/2} e^{-\theta_2/2}$$
$$X_6 = \rho_{13}\sigma_1\sigma_3 = \left(2e^{\theta_6}/(1 + e^{\theta_6}) - 1\right) e^{-\theta_1/2} e^{-\theta_3/2}$$
$$X_7 = \rho_{14}\sigma_1\sigma_4 = \left(2e^{\theta_7}/(1 + e^{\theta_7}) - 1\right) e^{-\theta_1/2} e^{-\theta_4/2}$$
$$X_8 = \rho_{23}\sigma_2\sigma_3 = \left(2e^{\theta_8}/(1 + e^{\theta_8}) - 1\right) e^{-\theta_2/2} e^{-\theta_3/2}$$
$$X_9 = \rho_{24}\sigma_2\sigma_4 = \left(2e^{\theta_9}/(1 + e^{\theta_9}) - 1\right) e^{-\theta_2/2} e^{-\theta_4/2}$$
$$X_{10} = \rho_{34}\sigma_3\sigma_4 = \left(2e^{\theta_{10}}/(1 + e^{\theta_{10}}) - 1\right) e^{-\theta_3/2} e^{-\theta_4/2}$$

Computing each $\dfrac{dX_j}{d\theta_i}$, $j, i = 1, \ldots, 10$, we obtain the Jacobian matrix of the transformation, which is lower triangular as $\dfrac{dX_j}{d\theta_i} = 0$, $i > j$. Then, only the diagonal elements are required to obtain the determinant, and they are given by

$$\frac{dX_j}{d\theta_j} = -e^{-\theta_j},\ j = 1, \ldots, 4,$$
$$\frac{dX_5}{d\theta_5} = \left(2e^{\theta_5}/(1+e^{\theta_5})^2\right) e^{-\theta_1/2}e^{-\theta_2/2}$$
$$\frac{dX_6}{d\theta_6} = \left(2e^{\theta_6}/(1+e^{\theta_6})^2\right) e^{-\theta_1/2}e^{-\theta_3/2}$$
$$\frac{dX_7}{d\theta_7} = \left(2e^{\theta_7}/(1+e^{\theta_7})^2\right) e^{-\theta_1/2}e^{-\theta_4/2}$$
$$\frac{dX_8}{d\theta_8} = \left(2e^{\theta_8}/(1+e^{\theta_8})^2\right) e^{-\theta_2/2}e^{-\theta_3/2}$$
$$\frac{dX_9}{d\theta_9} = \left(2e^{\theta_9}/(1+e^{\theta_9})^2\right) e^{-\theta_2/2}e^{-\theta_4/2}$$
$$\frac{dX_{10}}{d\theta_{10}} = \left(2e^{\theta_{10}}/(1+e^{\theta_{10}})^2\right) e^{-\theta_3/2}e^{-\theta_4/2}$$

Calculating the product of these terms and taking logarithms, the log-Jacobian is

$$\log\left|\prod_{j=1}^{10}\frac{dX_j}{d\theta_j}\right| = 6\log 2 - \frac{5}{2}\sum_{j=1}^{4}\theta_j + \sum_{j=5}^{10}\theta_j - 2\sum_{j=5}^{10}\log(1+e^{\theta_j}).$$

The other terms of the density $\pi(\boldsymbol{\theta}^*)$ correspond to the smoothing parameters λ_1. We consider a Uniform(0,100) prior distribution for the standard deviation $\sigma_{\lambda_1} = 1/\sqrt{\lambda_1}$, with density function $f(\sigma_{\lambda_1}) = 1/100$. The transformation is

$$\sigma_{\lambda_1} = \exp(-0.5 * \theta_{\lambda_1}) = g^{-1}(\theta_{\lambda_1}).$$

Then, the density function for θ_{λ_1} is

$$h(\theta_{\lambda_1}) = f(g^{-1}(\theta_{\lambda_1}))\left|\frac{dg^{-1}(\theta_{\lambda_1})}{d\theta_{\lambda_1}}\right| = \frac{1}{100}\left|\frac{-1}{2}e^{-\theta_{\lambda_1}/2}\right| = \frac{1}{200}e^{-\theta_{\lambda_1}/2}.$$

Then, the log density is

$$\log(h(\theta_{\lambda_1})) = \log(0.005) - \frac{1}{2}\theta_{\lambda_1}.$$

Within the "generic_mps_spat" function, this is included through the log.prior() function in this piece of code

```
########################################
## log.prior: return the log-prior for the hyperparameters
########################################
log.prior = function() {
   param = interpret.theta()
   ## Wishart prior for Covar
   sigma2 <- 1 # Wishart parameter
   val = log(MCMCpack::dwish(W = param$Covar, v = 2*k+1, S = diag(rep(sigma2, k))))
   ## sigma (1:J)
   val = val + sum(-(5/2)* theta[as.integer(1:k)])
   ## rho
   val = val + sum( log(2) + theta[as.integer( k+1:(k*(k-1)/2) )]  -
                  2*log(1 + exp(theta[as.integer( k+1:(k*(k-1)/2) )])))
   ## sigma lambda ~ Unif(0,100) - smoothing parameter
   val = val + sum(log(0.005)- (0.5)* theta[as.integer(k*(k+1)/2+1)])
   return (val)
}
########################################
```

C. Identifiability of correlation parameters

In this section, we show that the correlation parameters are identifiable. This is clear as $\rho_{\gamma jk} = \sigma_{\gamma jk}/(\sigma_{\gamma j}\sigma_{\gamma j})$, where $\sigma_{\gamma jk}$ is the covariance between the coefficients of the temporal P-splines of crimes j and k. Suppose that $\sigma_t^2 = 1/\lambda_3$ is subsumed in the between-crimes covariance matrix, denoted now by $\mathbf{\Sigma}_\gamma^*$. Clearly, the correlation parameters are identifiable as

$$\rho^*_{\gamma jk} = \sigma^*_{\gamma jk}/(\sigma^*_{\gamma j}\sigma^*_{\gamma j}) = (\sigma_{\gamma jk}\sigma_t^2)/(\sigma_t\sigma_{\gamma j}\sigma_t\sigma_{\gamma j}) = \rho_{\gamma jk}.$$

D. Succinct description of the crimes

In this section a brief description of the four crimes considered in the paper are provided.

Very succinctly, rape is committed if a "*man penetrates his penis, to any extent, into the vagina, mouth, urethra or anus of a woman or makes her to do so with him or any other person*" without her consent. Assault or criminal force to woman with intend to outrage her modesty includes sexual harassment, use of criminal force to a woman with intent to disrobe, voyeurism, and stalking. Cruelty by husband or husband's relatives refers "*to any conduct to drive the woman to commit suicide or to cause grave injury or danger to life, limb or health (whether mental or physical) of the woman*". It also includes "*harassment with the intention of coercing her or any person related to her to meet any unlawful demand for any property or valuable security...*". Finally, kidnapping and abduction comprises kidnapping in order to murder, kidnapping for ransom, or kidnapping to compel a woman to marry a person against her will. Given that the topic is very sensitive, for more information and details about the precise definition of these crimes, the reader is referred to the Indian Penal Code, sections 375 for rape, 354 (A, B, C, D) for assault, 498A for cruelty, and sections 364, 364A, and 366 for kidnapping and abduction.

E. Additional Figures for Subsection 4.2.2

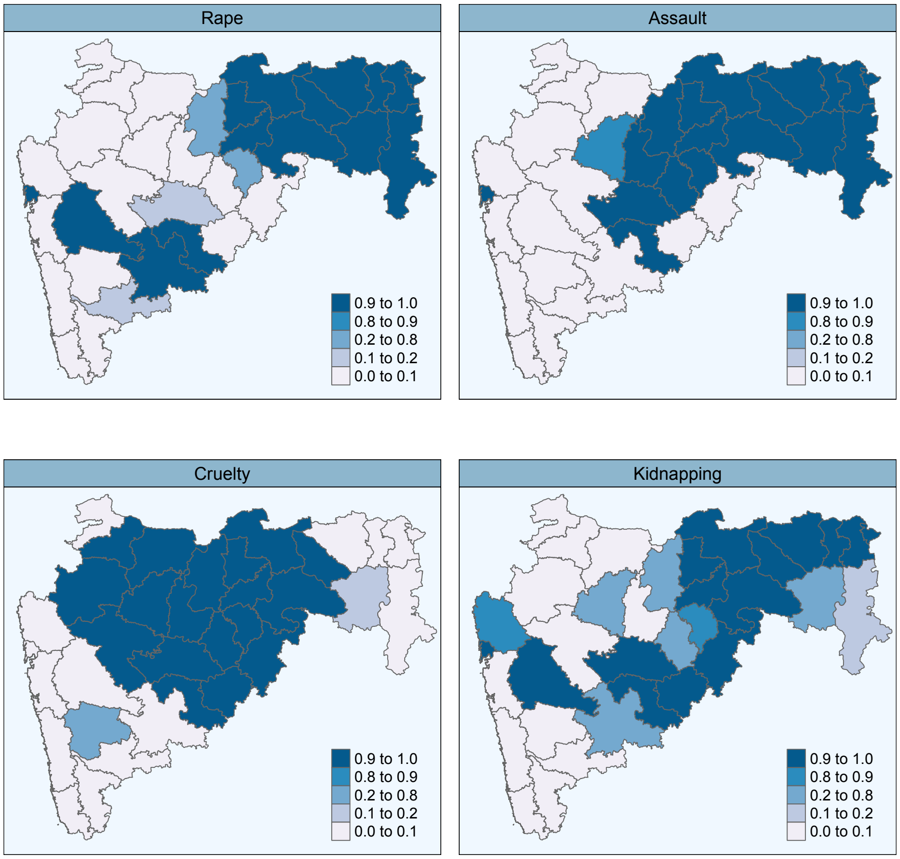

Fig. E.1. Exceedance probabilities for rape (top left), assault (top right), cruelty (bottom left), and kidnapping (bottom right).

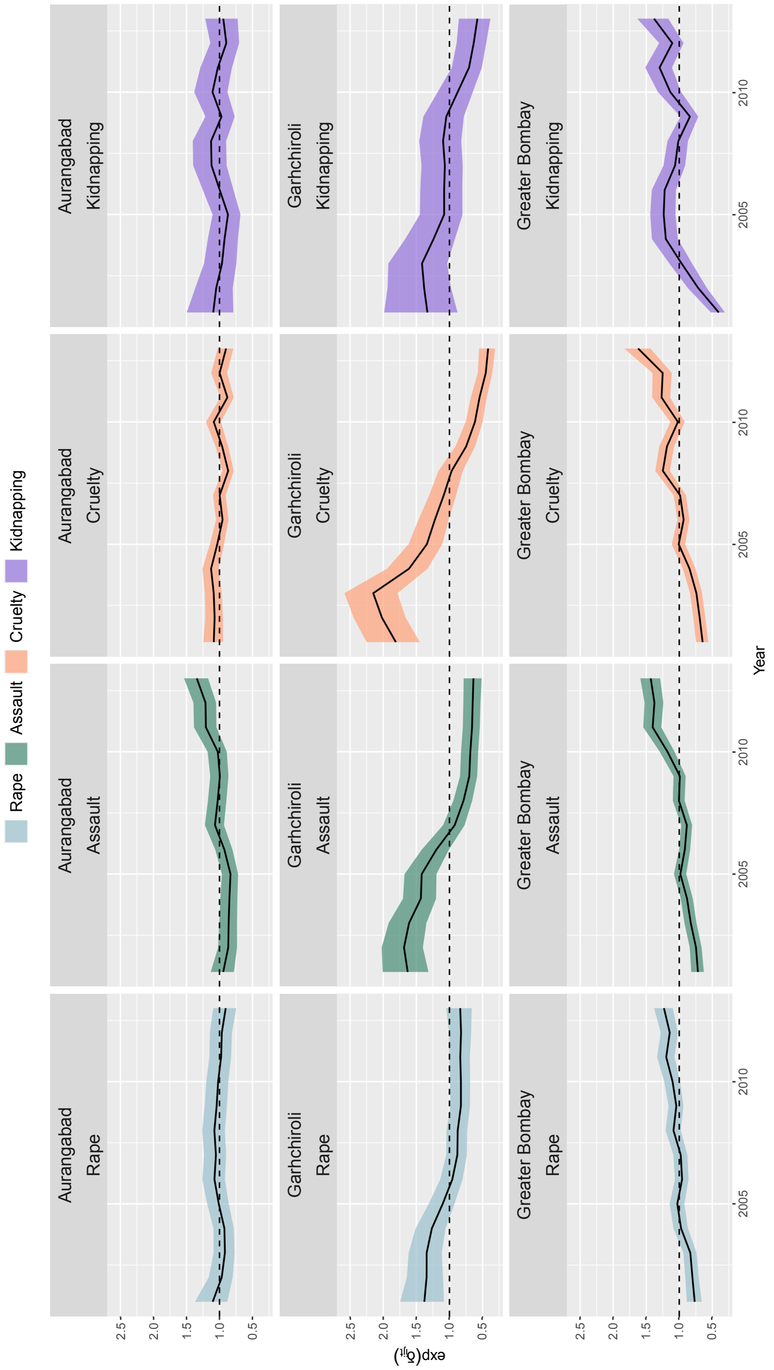

Fig. E.2. Specific temporal trends (posterior median of $\exp(\delta_{itj})$) for three selected districts: Aurangabad, Garhchiroli, and Greater Bombay.

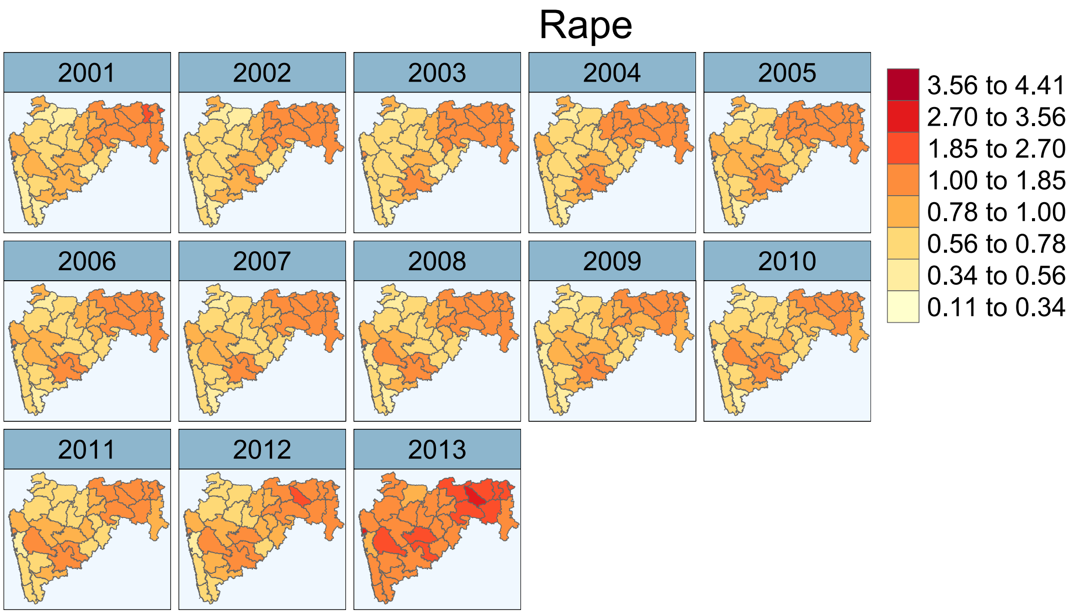

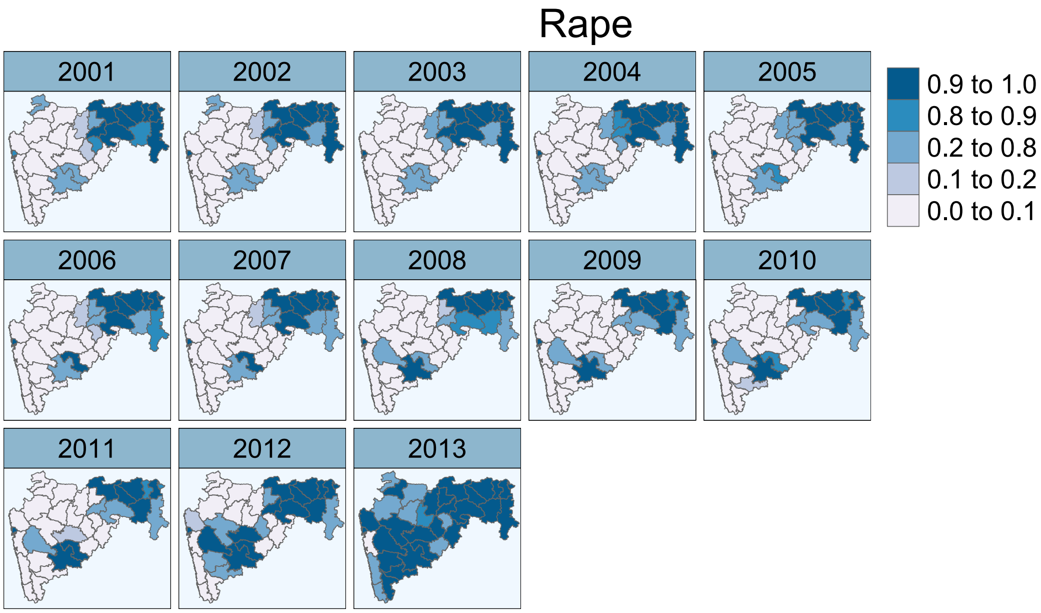

Fig. E.3. Map of estimated incidence risks for rape (top) and posterior probabilities that the relative risk is greater than one in Maharashtra between 2001 and 2013.

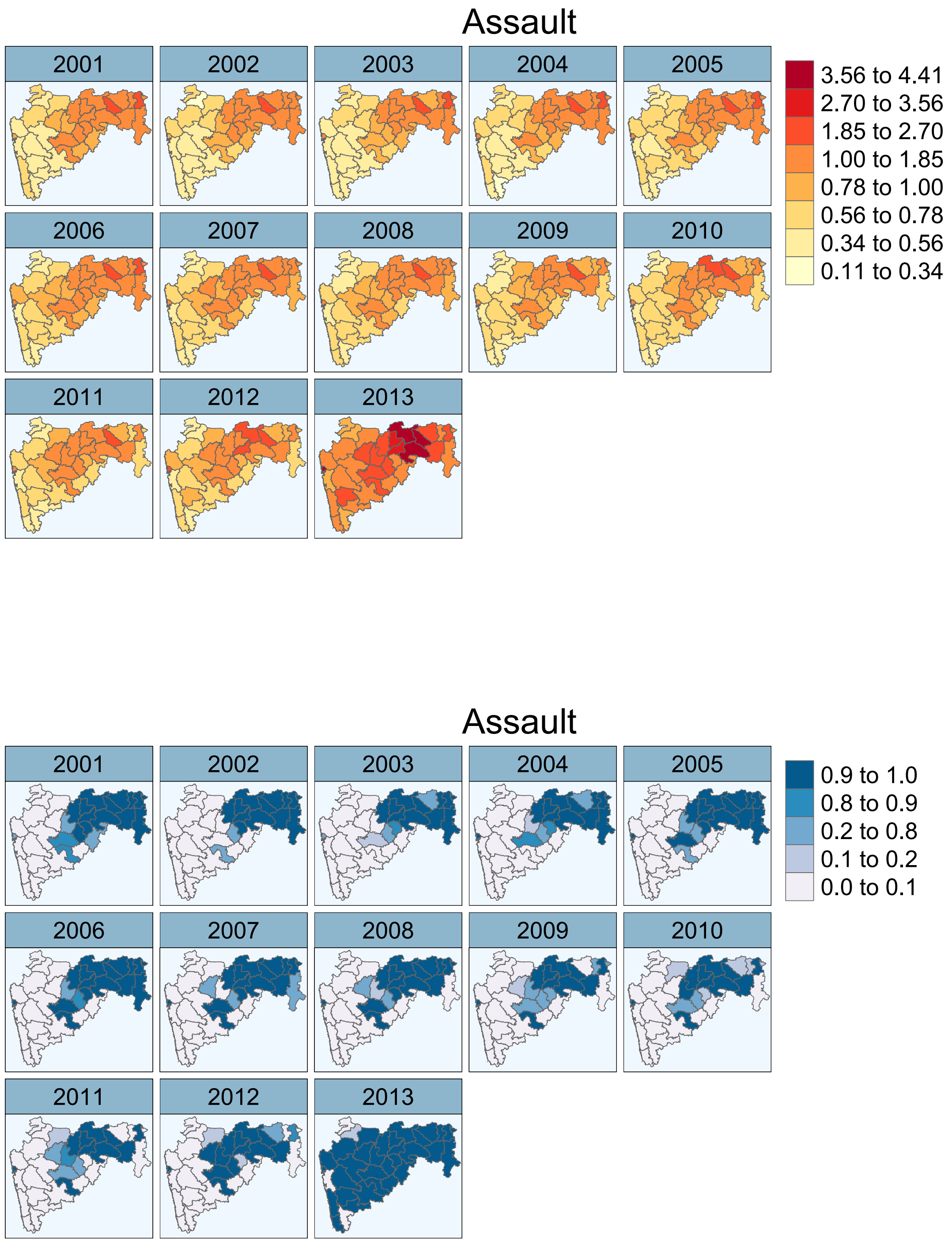

Fig. E.4. Map of estimated incidence risks for assault (top) and posterior probabilities that the relative risk is greater than one in Maharashtra between 2001 and 2013.

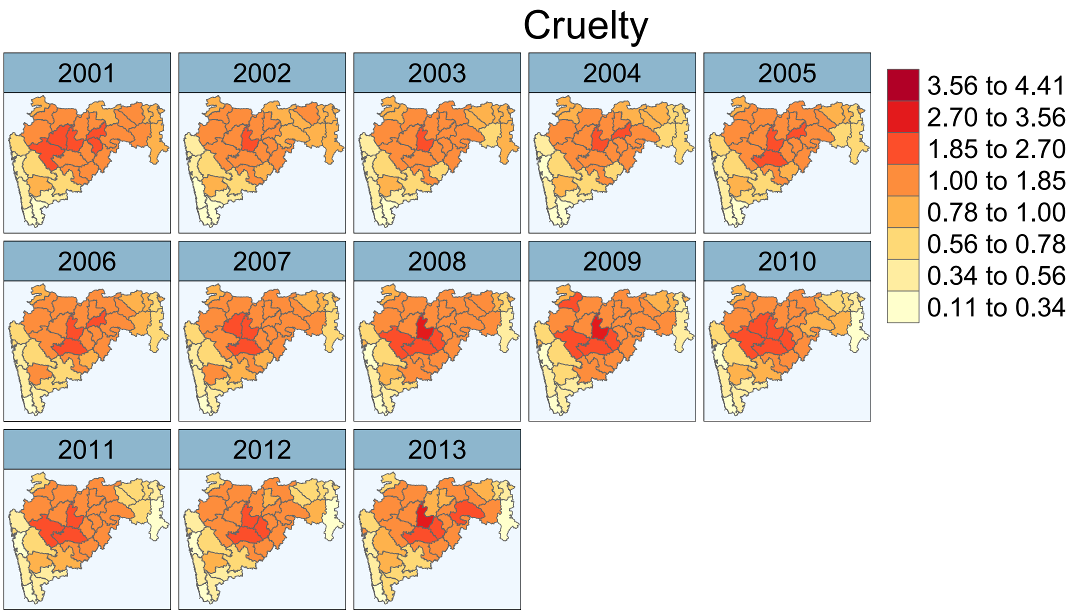

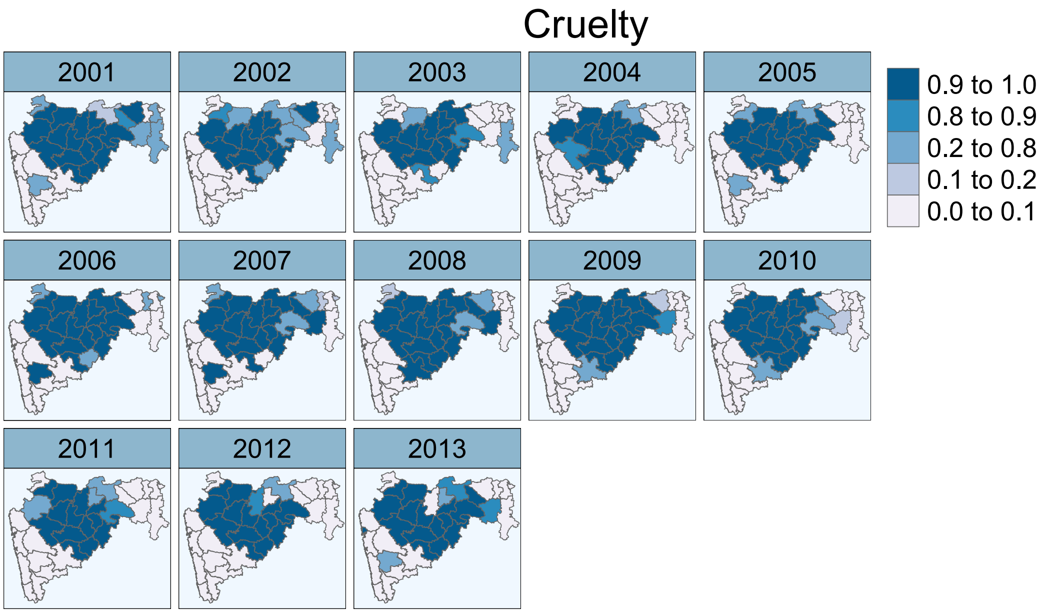

Fig. E.5. Map of estimated incidence risks for cruelty (top) and posterior probabilities that the relative risk is greater than one in Maharashtra between 2001 and 2013.

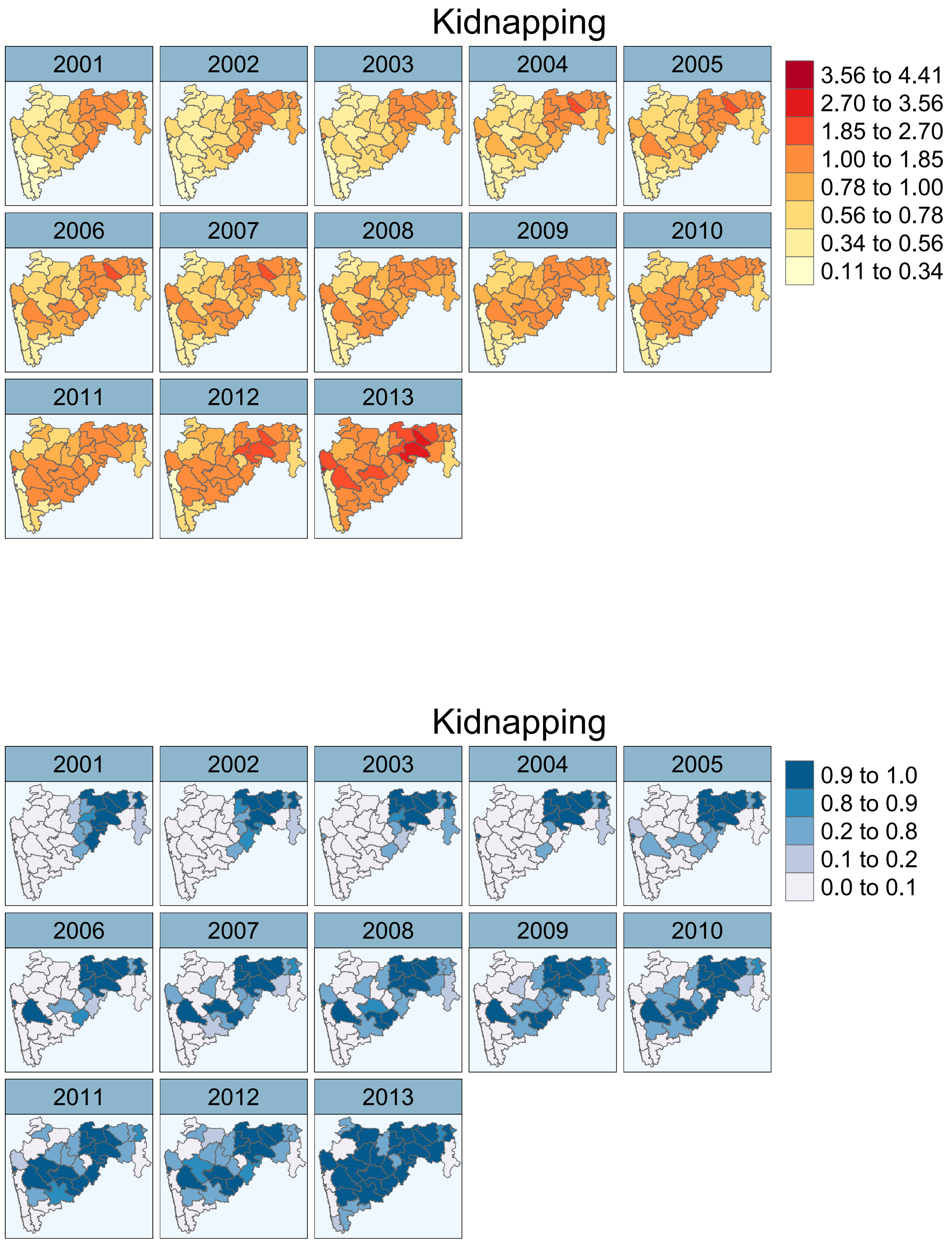

Fig. E.6. Map of estimated incidence risks for kidnapping (top) and posterior probabilities that the relative risk is greater than one in Maharashtra between 2001 and 2013.

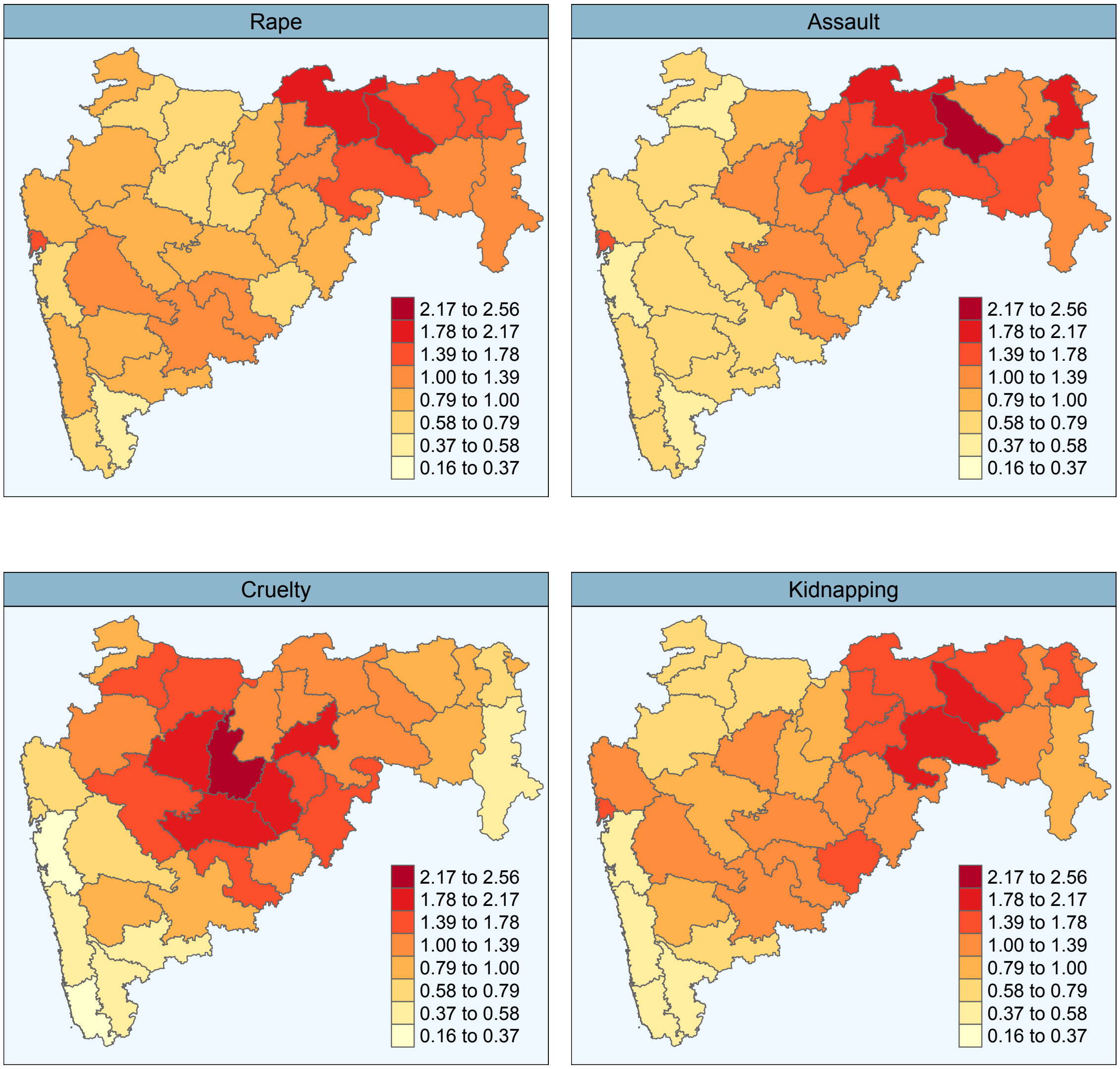

Fig. E.7. Posterior median of the district-specific spatial risk for rape (top left), assault (top right), cruelty (bottom left), and kidnapping (bottom right) obtained with the M-model

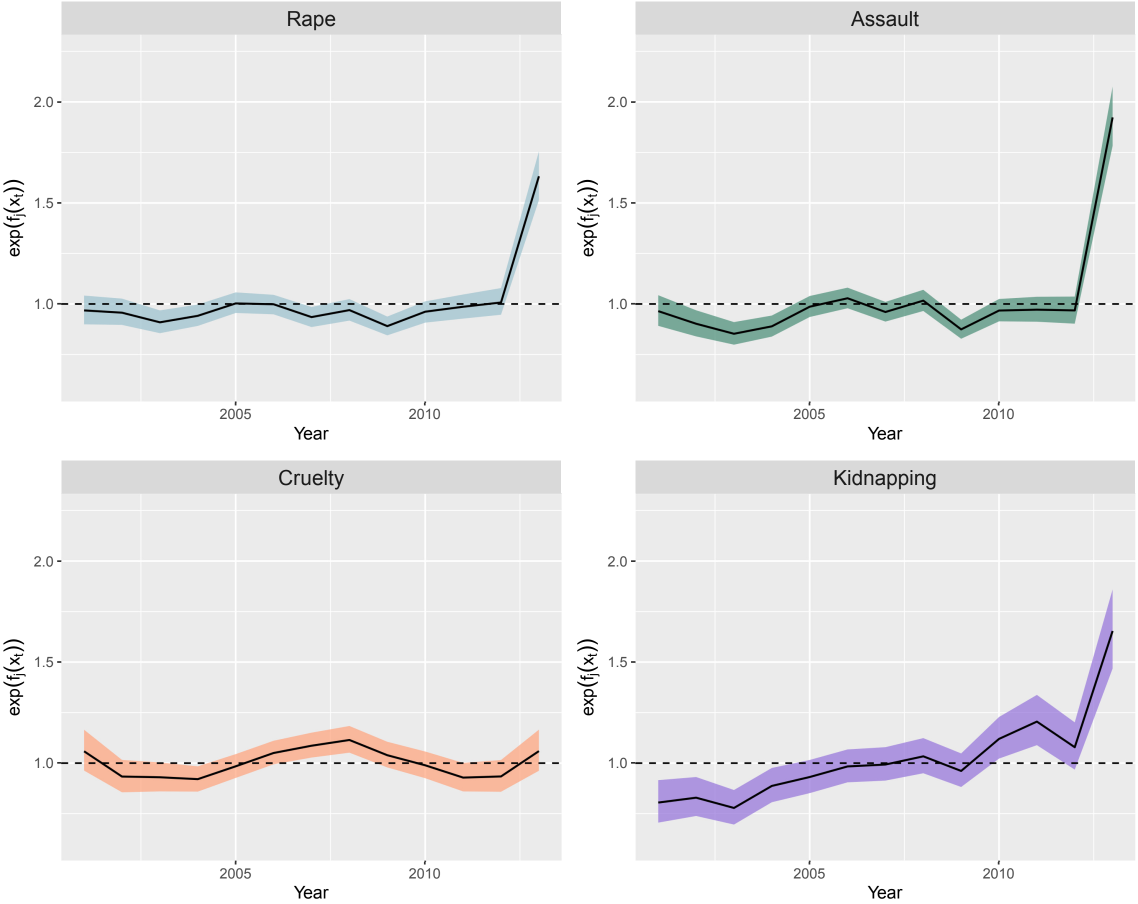

Fig. E.8. Temporal pattern of incidence risks for rape, assault, cruelty, and kidnapping obtained with the M-model
